# RNA-Seq Reveals Differentially Expressed Genes Associated with High Fiber Quality in Abaca (*Musa textilis* Nee)

**DOI:** 10.3390/genes13030519

**Published:** 2022-03-15

**Authors:** Nelzo C. Ereful, Antonio G. Lalusin, Antonio C. Laurena

**Affiliations:** 1 Philippine Genome Center for Agriculture, University of the Philippines Los Baños, Laguna 4031, Philippines; aclaurena@up.edu.ph; 2 Plant Physiology Laboratory, Institute of Plant Breeding (IPB), College of Agriculture and Food Science, University of the Philippines Los Baños, Laguna 4031, Philippines; 3 Institute of Crop Science, College of Agriculture and Food Science, University of the Philippines Los Baños, Laguna 4031, Philippines; aglalusin@up.edu.ph

**Keywords:** abaca, RNA-seq, differential expression, fiber quality, abaca bunchy top virus

## Abstract

Despite the importance of and current demand for abaca (*Musa textilis* Nee) fiber, there has been limited study that capitalizes on RNA-seq to identify candidate genes associated with high fiber quality and bunchy top virus (AbBTV) resistance. Three varieties (Abuab, Inosa, and Tangongon), one wild banana variety (*Musa balbisiana* Colla) Pacol, and two developed backcrosses (Abuab × Pacol BC_2_ and BC_3_) were grown at the Institute of Plant Breeding (IPB), Laguna, Philippines. The pseudostems of 3-month-old suckers of each genotype were sampled for RNA-seq. Datasets were analyzed for differential expression (DE) implementing various model frameworks, including pairwise, genotypic and non-DE models. Results indicate that Abuab and BC_3_ induce the highest proportion (70%) of abaca-specific genes. Gene ontology (GO) enrichment analysis showed several genes associated with cellulose synthase activity, callose synthase, ß-glucosidase activity, glucan biosynthetic process, etc. KEGG pathway analysis showed several genes encoding for enzymes involved in the lignin biosynthetic pathway. Analysis using genotypic DE (GDE) between abaca bunchy top virus (AbBTV)-resistant and -susceptible groups revealed genes such as pathogenesis-related protein and NBS-LRR. As the genotypes were not infected with the pathogen, these genes are yet to be confirmed for their roles in disease resistance and are an interesting subject for further investigation.

## 1. Introduction

Abaca (*Musa textilis* (L.) Nee), known internationally as Manila hemp, a close relative of banana (*Musa* sp.), is a leaf fiber, composed of long slim cells that form part of the leaf sheath. It is cultivated for its great mechanical strength, resistance to saltwater, and long fiber length.

Currently, the Philippines is the largest producer of abaca fiber in the world, supplying around 87% of global demand with production of 57,000 tons, followed by Ecuador with 10,000 tons [1]. The industry has contributed to the local Philippine economy with average annual earnings of PhP 4.7 billion (~USD 97 M). Right now, we have a supply deficit of 125,000 metric tons [2] due to increasing demand from textile and automobile industries, healthcare systems, etc. This has been compounded by a global shortage of personal protective equipment (PPE) in the medical sector brought about by the COVID-19 pandemic with orders coming non-stop [2]. Abaca demand is expected to further increase in the next few years due to fluctuating COVID-19 cases globally. Thus, it is a sought-after material in the healthcare industry because of its high medical grade. Abaca is also a raw material used to make ropes, fishing lines, currency notes, textile fiber, paper products including tea and coffee bags, among hundreds of other end-products.

Furthermore, demand for abaca is expanding in several industries. For example, there are now growing investigations into the possibility of using its fiber for aerospace and automobile industry applications [3]. In the car industry, composites reinforced with abaca fiber were found to exhibit better strength [4]. However, its hydrophilicity, which is attributed to numerous hydroxyl groups, imposes a drawback in these industries. Such a constraint provides an avenue for further research in both molecular biology and agricultural chemistry.

Abaca and banana are placed under the family Musaceae of the order Zingiberales. Abaca (T genome) is placed under the Australimusa section, members of which have a ploidy of 2n = 20 [5,6]. *M. acuminata* (A genome) along with *M. balbisiana* (B genome) are diploid but sometimes triploid. The double haploid Pahang belonging to *M. acuminata* subspecies *Malaccensis* has a ploidy of 2n = 22 with a 523-megabase genome [7]. Study further revealed that there are 36,542 protein-coding gene models in the *Musa* genome. On the other hand, Suthanthiram et al. [8] identified 35,783 unique transcripts in the *M. balbisiana* accession Attikol. Recently, the abaca variety Abuab was sequenced and was found to have an estimated length of 616 Mbp with 33,277 predicted gene structures [9].

Abaca, along with banana and plantain, are vegetatively propagated using suckers or tissue culture plants and are grown almost as perennial plantations. Abaca produces non-edible fruit and blooms from 24 to 30 months, at which time it is ready for fiber production [10]. There are 110 varieties in the southern parts of the Philippines (Mindanao) based on 25 qualitative and six quantitative morphological traits, as reported by [11].

Abaca contains 56–63% (wt.) cellulose, 20–25% hemicellulose, 7–9% lignin, and 3% waxes [12]. In a separate study, the biomass contents ranged from 11 to 21% regardless of the growth stages of abaca [13]. That study further revealed that pseudostem tissue showed the highest percentage of biomass (dry weight per plant) during the vegetative and flag-leaf stages of growth.

In the Philippines, local farming approaches remain rudimentary, with research platforms yet to be put in place to deal with pathogens such as abaca bunchy top virus (AbBTV) and abaca bract mosaic virus. A practical approach to control the virus is to employ phytosanitary measures and to use virus-free planting material [14].

Most abaca varieties have high fiber quality (FQ) but are sensitive to AbBTV. There are resistant lines identified from the abaca germplasm collection; however, these often have fibers of inferior quality [15]. Despite advances in molecular and computational biology, no study that capitalizes on RNA-seq—currently the method of choice to characterize trait-associated features on a genome-wide scale—has been performed to identify candidate genes associated with high fiber quality and AbBTV resistance.

In this study, we sequenced three different abaca varieties, one wild banana var. and two developed backcross lines (BC_2_ and BC_3_) using RNA-seq to shed light on the molecular underpinnings of high fiber quality and, to a modest extent, resistance against AbBTV. Using various models of differential expression (i.e., pairwise, genotypic, and non-differential expression models), we aimed to identify differentially expressed genes associated with fiber quality and AbBTV resistance. This information may supplement the research community’s continuing efforts in its pursuit to identify causative features linked to these important traits.

## 2. Materials and Methods

**Figure 1:**
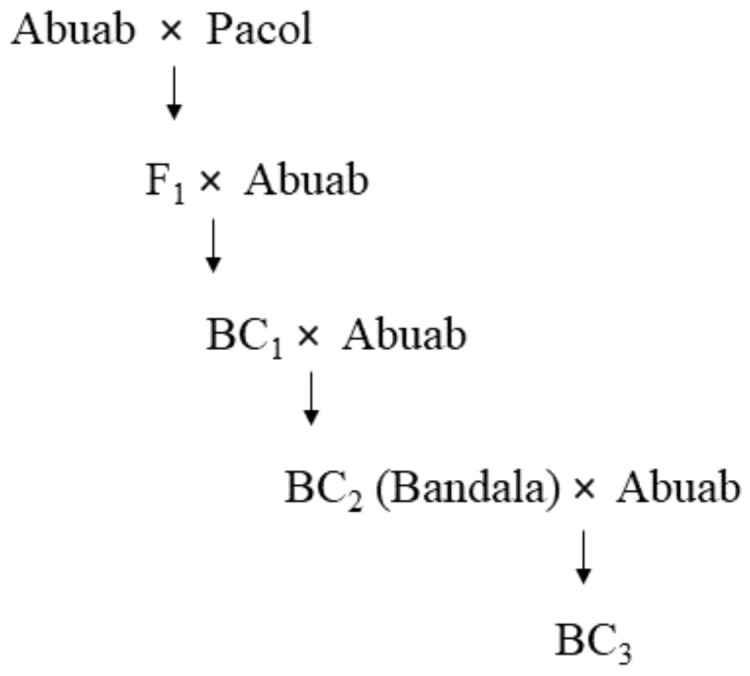
Schematic diagram of how backcrosses were generated. Abuab serves as a female parent recipient at the original stage of crossing (F_0_); Pacol, as male. In the succeeding backcrosses, Abuab serves as the recurring pollen donor, the source of high fiber quality alleles. Note that BC_2_ is nicknamed “Bandala”.

Three abaca varieties (Abuab, Inosa, and Tangongon), a wild indigenous banana variety (Pacol), and two developed backcross genotypes (BC_2_ and BC_3_) (described in Section 3 and Figure 1) were grown and maintained at the Feeds and Industrial Crop Section (FICS) collection site, located at the Institute of Plant Breeding (IPB), University of the Philippines Los Baños (14°09′09.7″ N, 121°15′39.2″ E). All of these varieties were exposed to the same field conditions. The three vars. Abuab, Inosa, and Tangongon were registered with the National Seed Industry Council (NSIC), Philippines, and are commercially grown due to their superior qualities [16]. Plant materials (3 biological replicates × 6 varieties) with uniform growth status (3 months old) were collected on 22 June 2021 between 10 and 11 AM. The innermost whorls of the midparts of the pseudostems were collected, snap-frozen in liquid N, and stored in −80 °C freezer until further extraction.

### 2.1. RNA Extraction

The three pseudostems representing three biological replicate-samples were pooled to make up to 1.0 g (~0.33 g each) from each variety in what were previously referred to as biologically averaged experiments [17]. It is more cost-efficient while maintaining statistical power [18]. Furthermore, pooling of samples is preferred over extracting each sample-replicate, as it is challenging to isolate RNA from abaca, as indicated by previous experiences of researchers and this paper’s authors. Pooling of samples has been performed in recent studies (e.g., [19,20]). Samples were ground using mortar and pestle with liquid N. Autoclavable equipment and reagents used were sterilized to avoid unwanted contaminants, including exogenous DNA, RNAse, microbes, etc.

RNA extraction was performed on these pooled biological samples using a modified cetyltrimethylammonium bromide (CTAB; with 2% ß-mercaptoethanol) method with overnight LiCl precipitation, according to the protocol described by the laboratory of Dr. John Carlson at the Schatz Center for Tree Molecular Genetics at Pennsylvania State University. All centrifugation steps were carried out under 4 °C to avoid RNA degradation. RNA was reconstituted using 800 µL SSTE with 1.5 µL Monarch^®^ DNAse in 10 µL DNAse buffer, incubated at 37 °C for 1 h. DNAse was removed by adding 24:1 chloroform:isoamyl. After spinning for 20 min at 14,000 rpm, the aqueous phase was obtained, and 0.1 volume of Na acetate and two volumes of absolute ethanol were added. Samples were incubated for 1 h at −80 °C, then were spun for 30 min at 14,000 rpm. The pellet was obtained and washed with 75% ethanol by spinning. Pellets were dried for 15 min, and 50-µL of nuclease-free water was added.

Bands were resolved in 2% agarose gels with 0.02 µL GelRed^®^ in Tris-acetic EDTA buffer using Mupid^®^, run under 110 V. Band images were viewed using Bio-Rad gel doc under UV transillumination with Image Lab software. Yield and RNA purity were assessed using a Biotek^®^ Epoch Microplate Spectrophotometer controlled with Gen5 software. Samples with 2.0 or greater at A260/230 and A260/280 were considered of good quality.

The pooled RNA samples of each of the six varieties were sent for RNA sequencing to Macrogen Korea with rRNA elimination (Ribo-Zero for plants). Additional quality assessment of the RNA samples was performed before library preparation and sequencing (see Table 1). TapeStation profiles of the six RNA samples extracted from the six genotypes were generated by the sequencing company (Supplementary Info S1). All samples passed the RNA quality evaluation prior to sequencing (Table 1).

Library preparation was performed according to the protocol followed by the company. Briefly, random fragmentation of the cDNA sample was performed, followed by 5′ and 3′ adapter ligation. Fragments were then PCR-amplified and purified. Libraries were loaded to a flow cell lane with surface-bound oligos complementary to the library adapters. RNA-seq was performed using Illumina Novaseq 6000, Paired-End (PE).

### 2.2. Bioinformatics Pipeline for Differential Expression

All command lines and R scripts used in this study are stored in our github container (https://github.com/nelcaster7/abaca_transcriptome_analysis, accessed on 25 January 2022). All computational analysis was performed using Linux environment (Debian 4.9.246-2); statistical analysis was performed using Rstudio v.1.4.1717 [21].

#### 2.2.1. Reads Pre-Processing

Quality checking of the reads was performed using FastQC [22]. Due to the absence of low-quality bases and adapter sequences, no cleaning procedure was necessary.

#### 2.2.2. Mapping

Abuab PE reads were aligned against the recently published Abuab reference assembly [9] using STAR v.2.7.7a [23] allowing no mismatches (--outFilterMatchNmin 0). After experimenting on other arguments, we used the following options, yielding a high percentage of uniquely mapped reads: --outFilterScoreMinOverLread 0.3 --outFilterMatchNminOverLread 0.3, with all other arguments set to default. Similarly, Inosa, Tangongon, and Pacol PE reads were aligned using the same tool and parameters, except that for Pacol, we used the *M. balbisiana* double haploid of Pisang Klutuk Wulung (DH–PKW) as genome reference sequence [24]. All alignment output was converted to BAM and sorted using SAMtools [25] (Li et al., 2009).

Likewise, because BC_2_ and BC_3_ are mostly Abuab (87.5% and 93.75%, respectively), we initially aligned the reads against the Abuab reference assembly allowing no mismatches. Results, however, showed low mapping rates. As the backcrosses harbor Pacol-specific alleles, unmapped reads were re-mapped against the *M. balbisiana* genome assembly. Data counts were used for the GDE model between resistant and susceptible varieties (see Results and Discussion Section 3.2.4).

#### 2.2.3. Read Count Quantification

Read count quantification in a BAM alignment file was performed using the featureCounts program from the subRead package [26] against a GTF. We counted the primary alignment alone (option: --primary) with the following additional arguments: -t exon, the feature type to count read against, and -g transcript_id, the attribute type to summarize counts as recommended by [27].

#### 2.2.4. Differential Expression (DE)

We performed DE using bayseq [28,29] between and among the varieties. Because abaca (*M. textilis*) and Pacol (*M. balbisiana*) belong to two different species, we first determined putative orthologs using the webtool OrthoVenn2 [30] implementing an Expectation value of 1 × 10^−5^ and an Inflation index of 1.5 with the peptide sequences of each reference variety as Fasta input sequence.

Orthologous transcripts between abaca and Pacol have varying sequence lengths. Therefore, we provided baySeq with length information of each feature of each genotype using the “seglens” slot. Hence, two matrices were fed to bayseq: (1) seglens, which contains the transcript length of each feature for each variety, and (2) a raw data count of all *Musa* sp. corresponding to the number of reads mapping to the reference assemblies. To normalize read counts, library scaling factor was calculated using Trimmed Means of M-values (TMM [31]) which accounts for sequencing depth, and transcript length.

DE was carried out by implementing various model frameworks including pairwise and more complex models following the instructions as previously described [28,29]. Pairwise differential expression (DE) was implemented to capture genes significantly differentially expressed between an abaca variety (high FQ) and Pacol (low FQ). Transcript isoforms with nearly zero row sums (≤2) in the data matrix were removed to increase the computational speed and decrease memory size of the data object (e.g., Abuab count + Pacol count > 2 were retained for analysis; R command: rowSums(CD@data) > ncol(CD)). We assigned Pacol as the lone group with a low FQ, and the rest (abaca) as another group with a high FQ.

To evaluate whether the removal of low read counts was effective, histograms and summary statistics were generated. Correlation matrix was also generated using the normalized counts of each variety. MA plots were created (Figures S2–S6; discussed in the Results and Discussion) to determine whether normalization was effective.

More complex models were implemented to identify genes between two groups. For genotypic DE (GDE) for FQ, abaca varieties were classified as one group and Pacol as another group. For non-differential expression (NDE), all abaca varieties (Pacol was dropped) were grouped as one to identify transcripts commonly significantly expressed. For GDE between resistant (R) and susceptible (S) varieties, Abuab, Inosa and Tangongon were classified as S, and Pacol and the backcrosses as the R group. As the resistance of the backcrosses was conferred by Pacol, we re-mapped the unaligned sequences to the *M. balbisiana* DH–PKW reference assembly using the same tool and parameters (uniquely or primary alignment only). These backcross data counts generated from the remapping, along with Pacol data count, comprised the R group. We then generated an MA plot to determine if normalization was effective (discussed in Section 3.2.4).

Bayseq calculates posterior likelihoods and generates normalized read counts. For PDE, expression ratios were calculated as Abaca/Pacol incremented with a pseudo count of 1 to avoid 0 denominators. Log (base 2) ratio or fold change (FC) was then computed. In PDE analysis, a gene (or a transcript ortholog) is said to be differentially expressed if it exhibits |log2FC| ≥ 1 (or FC ≥ 2), false discovery rate (FDR) p-value correction < 0.05 [32], and a total normalized read count of at least 20 reads to minimize the introduction of artifacts and ensure that the genes are expressed. The use of the total parental read count threshold of 20 has been implemented in several papers (e.g., [33,34]). For the more complex models, we considered genes as differentially expressed if they exhibited an FDR < 0.05.

#### 2.2.5. Gene Ontology (GO) Enrichment Analysis

GO enrichment analysis was performed using OmicsBox v.2.0.29 [35] by BLAST2GO implementing Blastx-fast program specifying non-redundant protein sequences (nr v5) and Musacea (*Musa* sp.) as taxonomic filter. BLAST expectation value was set at 1 × 10^−5^, with the rest of the arguments set at default.

Pathway analysis was performed using Kyoto Encyclopedia of Genes and Genomes (KEGG [36]) available online with *Musa acuminata* and monocots as subject database with BBH (bi-directional best hits) as Assignment Method turned on.

### 2.3. Ethical Standards on the Use of Plant Materials

We comply with the highest standards of institutional and national protocols on the use of biological materials. All plant materials used in this study were neither endangered nor at risk of extinction. Therefore, no ethical implications were associated with the use of such materials.

### 2.4. Availability of Data and Materials

All datasets have been uploaded in EMBL-EBI ArrayExpress (www.ebi.ac.uk/arrayexpress, accessed on 12 January 2022) with an assigned accession number: E-MTAB-10990.

## 3. Results and Discussion

All *Musa* sp. varieties used in this study were grown and maintained at the FICS site of the Institute of Plant Breeding (IPB), UP Los Baños. The innermost whorls of the midsection of three abaca varieties (*M. textilis* vars. Inosa, Tangongon and Abuab), two backcrosses, and one wild indigenous banana variety (*M. balbisiana* var. Pacol) (Table 2) were harvested in their vegetative stage (3-month-old suckers), a phase when they are actively growing. For brevity and simplicity, since the genotypes BC_2_ and BC_3_ lines are composed mostly of Abuab alleles (Table 2), we classified them as abaca genotypes, and *M. balbisiana* (var. Pacol) as banana.

The phenotypic performance of these varieties, as observed by previous researchers [15,37,38,39,40,41,42] is summarized in Table 2. Apparently, all abaca varieties have superior fiber quality (FQ) but are susceptible to AbBTV; Pacol has low FQ but has high resistance against AbBTV.

**Table 2:** Phenotypic performance of the six different *Musa* sp. varieties.

Varieties	Resistance/Susceptibility vs. AbBTV	Fiber Quality	References
Abuab (*M. textilis*)	S	High	[38]
Inosa (*M. textilis*)	S	High	[38,39]
Tangongon (*M. textilis*)	S	High	[40]
BC_2_ (87.5% Abuab; 12.5% Pacol)	R	High	[15,38,41,42]
BC_3_ (93.75% Abuab; 6.25% Pacol) *	R	High	
Pacol (*M. balbisiana*) (wild banana)	R	Low	[38]

* BC_3_ was observed to have high resistance against AbBTV and high fiber quality based on initial field assessment.

The backcrosses BC_2_ (locally dubbed as “Bandala”, which stands for Backcross Abaca with Native and Desirable Accessions to Lift Up the Abaca Industry), and BC_3_ were generated by crossing Abuab and Pacol. Their F_1_ hybrid was subsequently backcrossed to Abuab as the recurring pollen donor (see Figure 1). Both backcrosses have high fiber quality and thus carry Abuab’s phenotypic character and are AbBTV-resistant, a trait conferred by Pacol.

### 3.1. RNA-Seq Data Information

Briefly, three biological samples with the same weight (~0.33 g) were pooled to make up to 1.0 g from each variety. Total RNA was extracted for RNA-seq with ribosomal RNA removal (Ribo-Zero) using Illumina Novaseq 6000, Paired-End (PE) (see Section 2).

Results of quality checking showed the absence of low-quality bases and adapter sequences. Therefore, no further pre-processing step was required. Information on the sequencing reads of each variety are shown in Table 3. A total of 217,956,486 PE reads (2 × 101 bp) were generated across all genotypes collected.

### 3.2. DE between and among the Varieties

Reads generated from the abaca varieties were mapped to the Abuab scaffold assembly [9], and Pacol to the *M. balbisiana* DH–PKW [24]. Alignment yielded relatively high mapping rates for the three varieties—Abuab, Inosa, and Tangongon—with more than 80% (Table 4). Pacol had relatively low unique mapping (43.10%) and high multi-mapping (32.8%) rates, which were attributed to its highly repetitive sequences [24].

BC_2_ and BC_3_ yielded relatively low uniquely aligned reads with 59.96% and 65.37% mapping rates, respectively, since they are hosts to another genome—the Pacol genotype. BC_3_ had a higher mapping rate than BC_2_, which was ascribed to the higher proportion of Abuab alleles in its genome. The unmapped reads were subsequently re-mapped against the *M. balbisiana* DH–PKW genome assembly for GDE of AbBTV resistance (see Section 3.2.4).

Differential expression (DE) was performed using baySeq to capture segments that are differentially expressed between and among varieties. Because Pacol and the abaca varieties are inter-specifically different, ortholog searching between the total peptide sequences of Abuab and DH–PKW reference assemblies was performed using OrthoVenn [30] before DE (see Section 2). Results showed that there were 13,991 orthologous cluster groups between the two reference sequences with 32,935 combined protein abundance. Of these, 16,403 belonged to Abuab (48.7%), and 16,892 (51.3%) belonged to *M. balbisiana* DH–PKW (Figure S1).

There are a total of 27,610 annotated transcripts in Abuab and 33,021 in DH–PKW [9,24]. We found 14,859 unique (i.e., not duplicated) orthologous transcripts between Pacol and the abaca varieties (unmatched transcripts were dropped from DE as previously performed) [43].

**Figure 2:**
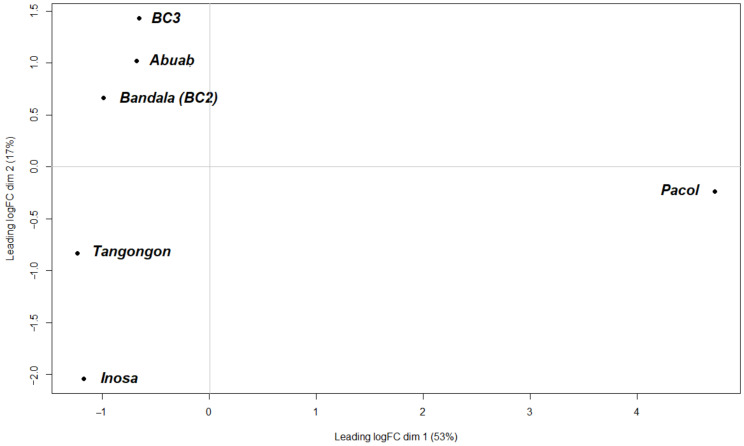
MDS plot of the six *Musa* sp. using normalized RNA-seq data counts.

All DE steps were carried out with a normalization procedure to remove biases due to varying library sizes and varying segment lengths. Prior to DE, a multi-dimensional scaling (MDS) plot was generated using the normalized datasets to identify relationships among the samples (Figure 2). Dim. 1 (*x*-axis) of the log-transformed datasets largely explained the variations between the two groups—abaca and Pacol (a *M. balbisiana* variety)—with the latter occupying the far-right side of the plot, signifying its divergence from the *M. textilis* varieties. Abuab and its backcrosses (BC_2_ and BC_3_) were clustered on the upper quadrant of dimension 2 (*y*-axis), which underscored their genetic relatedness. Inosa and Tangongon aggregated on the lower section of the *y*-axis, a departure from the Abuab and the Abuab-derived backcrosses.

**Figure 3:**
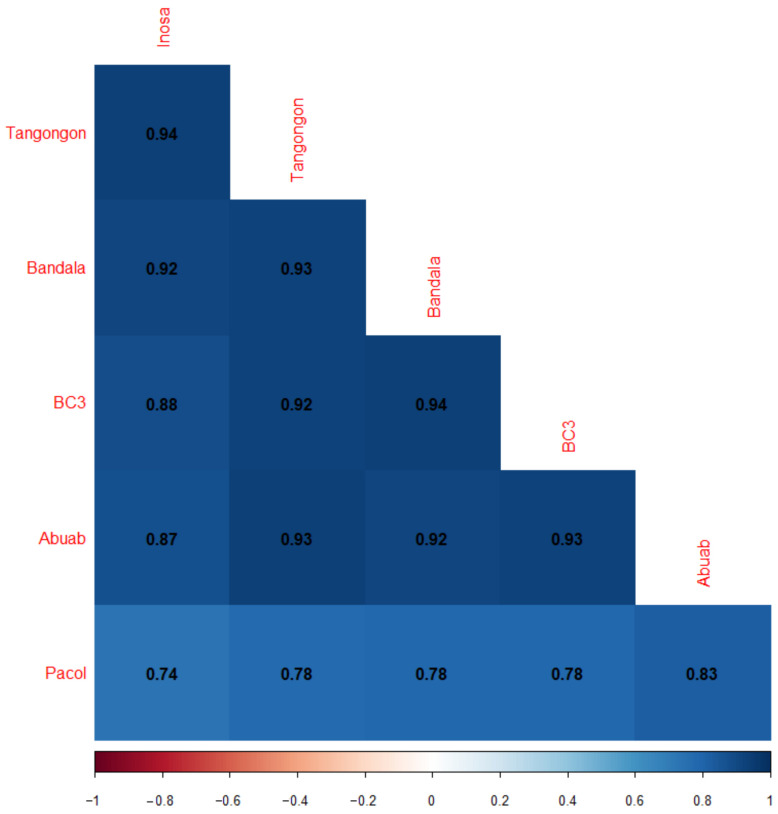
Correlation matrix among the six *Musa* sp. Color scale bar is shown below the matrix.

The inter-specific expression divergence of Pacol was further highlighted by the correlation matrix (Figure 3) generated using the normalized read counts. Correlations among the five abaca varieties were evidently high, emphasizing their intra-specific relationships.

#### 3.2.1. Pairwise Differential Expression (PDE)

Pacol was the lone variety with inferior FQ but high AbBTV resistance. Therefore, we performed DE between this variety and each abaca genotype to identify potential candidate genes for superior FQ. BC_2_ and BC_3_ were initially aligned against the Abuab reference; therefore, reads were Abuab-specific segments, which may confer high FQ. All MA plots for PDE showed symmetrical data points (at M = 0), which suggested that the normalization step was effective in correcting sequencing and transcript length biases (Figures S2–S6).

**Figure 4:**
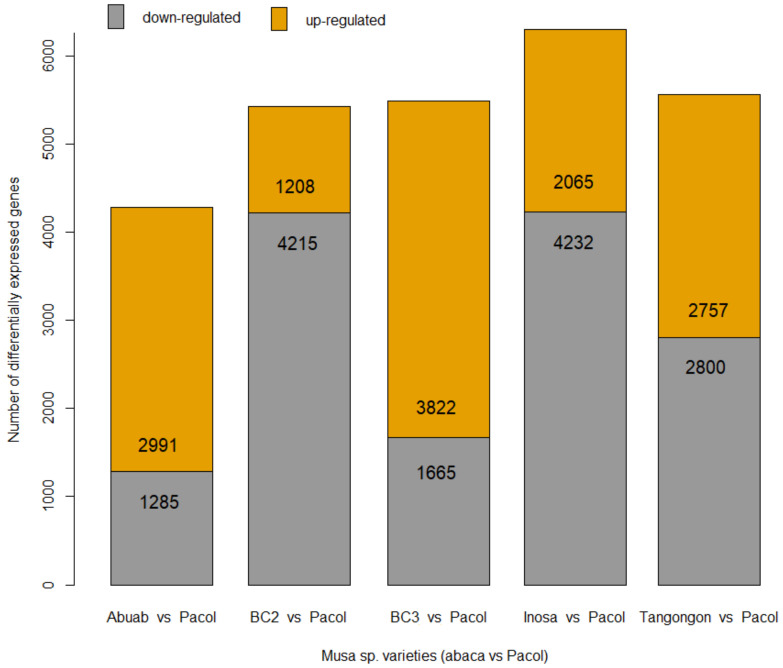
Number of up- and down-regulated DEGs (or transcript orthologs) between each abaca variety and Pacol.

PDE between Abuab and Pacol yielded 4276 transcript orthologs significantly differentially expressed (FC ≥ 2; FDR < 0.05; see Methods for complete statistical analysis). Of these, 2991 were up-regulated in Abuab (70% of the total differentially expressed genes (DEGs), while 1285 were down-regulated (Figure 4) (see Table S1 for complete list of PDE genes between Abuab and Pacol; Figure S2 for MA plot). Abuab yields best and is well-adapted to Luzon, the northern region of the archipelago [40,44] (as cited in [9]) where the samples were grown and collected. Interestingly, BC_3_, which harbors 94% Abuab alleles, also showed 70% transcript orthologs (3822) being up-regulated over the total number of DEGs (5487) (Table S2; Figure S3 for MA plot). Abuab and BC_3_ showed high proportions of up-regulated genes, which may be ascribed to their adaptation to the regional site and suggestive of their suitability for fiber production in Luzon.

Surprisingly, Bandala (BC_2_) demonstrated the lowest proportion of genes up-regulated: 1208 (22.3% of 5423) (Table S3; Figure S4 for MA plot). A large number of abaca- (Abuab-) specific genes potentially linked with superior fiber quality were down-regulated. We speculate that, as BC_2_ is highly heterozygous, the interactions between the two co-residing genomes (Abuab- and Pacol-specific alleles) as influenced by environmental conditions, can largely affect regulation of the expression of its alleles, as previously shown in other crops [45,46]. Bandala is also grown in several areas in Visayas, the central region of the Philippines, and in Mindanao, besides Luzon [42]. This indicates its ability to adapt to multiple environmental conditions owing to its heterozygous alleles, which confer it with transcriptional versatility or heterotic malleability, as recently shown in rice hybrids [47].

DE between Inosa and Pacol exhibited the highest number of differentially expressed transcript orthologs (6297) (Figure 4; Table S4; Figure S5 for MA plot). However, the numbers of up-regulated transcripts were relatively low (32.8%), potentially because this variety is more adapted to Visayas, the central region of the Philippines, and not Luzon [40,44] as cited in [9].

Tangongon, on the other hand, is well-adapted to Mindanao, the southern region of the country. There were 5557 transcript orthologs that were differentially expressed between the two varieties, with an almost equal number of genes (~50%) being up- and down-regulated (Table S5; Figure S6). Early and recent reports in the literature suggest an enormous influence of environmental conditions on the expression of genes (G × E). We, therefore, hypothesize that the number of induced genes in Tangongon may rise if it is grown in its native habitat, i.e., Mindanao, where it expresses its full (abaca) potential. We suggest that a similar study be performed in the southern area.

**Figure 5:**
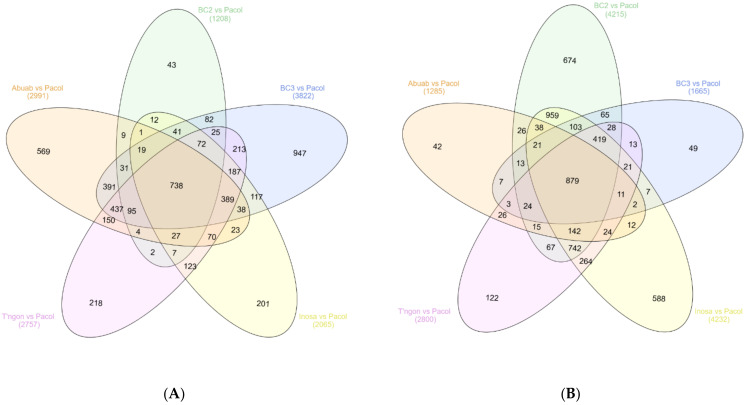
Commonly (**A**) up- and (**B**) down-regulated transcript orthologs between abaca variety and Pacol. Each ellipse represents the number of differentially expressed transcript orthologs between an abaca genotype and Pacol. Figure generated using InteractiVenn [53].

After DE between the abaca varieties and Pacol, we generated Venn diagrams to identify genes commonly up- and down-regulated. Results showed that there were 738 transcripts orthologs commonly induced in the five abaca varieties that were down-regulated in Pacol (Figure 5A; Table S6 for list of transcript isoforms). On the other hand, there were 879 transcript orthologs commonly repressed (or down-regulated) in the abaca varieties but up-regulated in Pacol (Figure 5B; Table S7).

**Figure 6:**
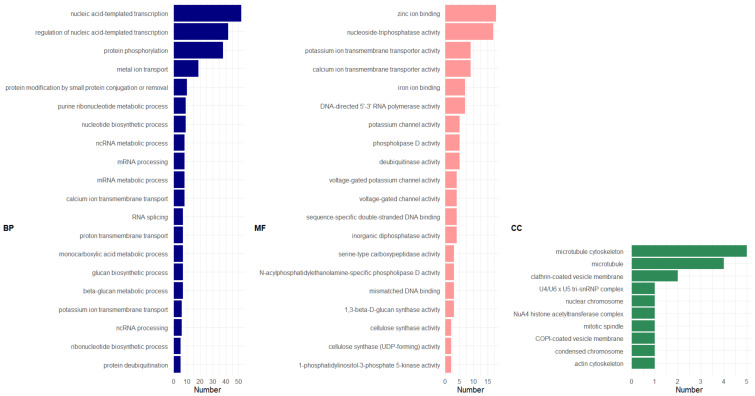
GO distribution of genes up-regulated in the abaca varieties but down-regulated in Pacol using PDE model for FQ. Legend: BP, Biological Process; MF, Molecular Function; CC, Cellular Component. Number indicates number of sequences or genes classified in each GO term. Figure generated using ggplot2 [57] in Rstudio [21]. GO distribution analyzed using OmicsBox [35].

GO enrichment analysis of the 738 genes commonly up-regulated (overlapping section in Figure 5A) suggests that transcript orthologs associated with nucleic acid-templated transcription and its regulation (GO:0097659; GO:1903506) were the most frequently enriched biological process (BP; Figure 6; also see Figure S7 for graphical representations of BP enrichment). Since abaca varieties are mostly made up of cellulose and hemicellulose [48], as reviewed in [49], we identified genes associated with polysaccharide synthesis. Several genes encoding for putative cellulose synthase A (LOCUS_024375-RA; LOCUS_001166-RA) and callose synthase (LOCUS_000059-RA; LOCUS_005801-RA; LOCUS_005802-RA) were found to be significantly enriched and were associated with both the glucan biosynthetic process (BP; GO:0009250) and ß-glucan metabolic process (GO:0051273) (see list of genes categorized in each GO term in Table S7).

KEGG pathway analysis of genes differentially expressed (up-regulated in abaca varieties) showed the participation of LOCUS_003933-RA (Dolichyl-phosphate β-glucosyltransferase) in the N-Glycan biosynthetic pathway. The gene catalyzes the initial transfer of a glycan group [50], but its function in abaca fiber synthesis is yet to be known. Additionally, KEGG pathway analysis further showed the enrichment of genes associated with the biosynthesis of secondary metabolites, one of which is shikimate O-hydroxycinnamoyltransferase (LOCUS_014669-RA), a key enzyme in lignin biosynthesis [51]. Modification of this gene through RNA interference causes reduction of lignin levels in *Brachypodium distachyon* [52].

The GO term cellulose synthase activity (molecular function (MF); GO:0016759, GO:0016760) (see Figure S8 for graphical representation) was, likewise, significantly enriched. Included in this GO category are the genes LOCUS_024375-RA and LOCUS_001166-RA, described above. Further GO analysis showed that microtubule, microtubule cytoskeleton, and actin cytoskeleton were significantly enriched Cellular Component categories (GO:0015630, GO:0005874, GO:0015629, respectively; Figure S9). Included in these terms are LOCUS_009196-RA which encodes for microtubule-associated protein TORTIFOLIA1; LOCUS_000975-RA and LOCUS_006594-RA, for kinesin-like protein. Microtubules and actins play essential roles during cell division and expansion (reviewed in [54]). These cellular components have been recently shown to be involved in fiber synthesis in cotton [55] and flax [56].

Similarly, we performed GO enrichment analysis of the 879 transcript orthologs up-regulated in the variety Pacol (or down-regulated in the abaca varieties). Results of GO distribution and the GO enrichment table are shown in Figure S10 and Table S8, respectively. However, as we were only interested in finding genes associated with FQ in abaca, they are not discussed in this paper exhaustively.

#### 3.2.2. Genotypic Differential Expression between *Musa* Groups

We implemented a more complex model framework, which we referred to here as genotypic differential expression (GDE) for FQ. Here, all abaca varieties (with high FQ) were assigned as one group, and Pacol (with low FQ) as a lone, separate group. While the aim was similar to PDE in finding potential causative features, this model was implemented to further mine for candidate genes associated with high FQ that were not captured by PDE. MA plot showed a symmetrical data cloud between the two groups (Figure S11).

Results showed that there were 1325 transcript orthologs differentially expressed between the two *Musa* groups (FDR < 0.05) (see Table S9). Of these, 575 were up-regulated in the abaca group and 750 in the Pacol variety group. Comparing the two approaches (PDE and GDE) for genes up-regulated in abaca (excluding those down-regulated), results indicated that a small proportion (58) are commonly induced between the two models (PDE vs. GDE), while 517 were identified uniquely by GDE, and 680 by PDE.

**Figure 7:**
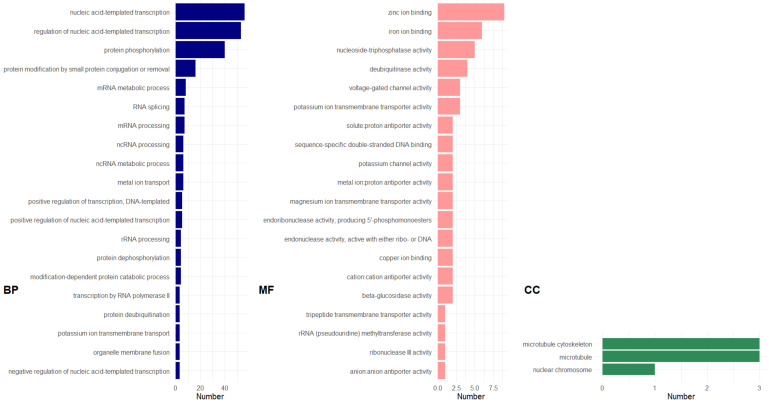
GO distribution of genes up-regulated in the abaca varieties but down-regulated in Pacol using GDE model for FQ. Legend similar to Figure 6. Figure generated using ggplot2 [57] in Rstudio [21]. GO distribution analyzed using OmicsBox [35].

GO enrichment of the 575 up-regulated GDE genes showed enrichment of genes associated largely with a range of nucleic acid and protein processing (BP; Figure 7; see Figure S12 for graphical representation; Table S10 for list of genes classified under each GO category). The GO terms cation (copper, zinc, and iron) binding and ß-glucosidase activity were found to be significantly enriched for MF (Figure S13). The latter included LOCUS_010531-RA and LOCUS_007060-RA, both encoding for glucan endo-1,3-ß-glucosidases. KEGG pathway analysis showed that both genes are involved in starch and sucrose metabolism.

Further results showed only three cellular component (CC) terms, which included microtubule, microtubule cytoskeleton and nuclear chromosome. These CC transcripts were also found in the PDE GO analysis, strongly suggesting their participation in fiber synthesis (Figure S14).

#### 3.2.3. Non-Differential Expression (NDE) Model across Abaca Varieties

Another model framework we implemented was the NDE model, which captures genes commonly and significantly expressed and associated with high FQ across the abaca varieties. This excluded Pacol, thus, eliminating the need to find DEGs between two contrasting genotypic groups. It also allowed inclusion of all abaca transcript isoforms for NDE assessment without the need to identify orthologous transcripts.

Results revealed that 3737 of the 20,787 genes (18%) were significantly expressed across the five abaca varieties (FDR < 0.05). Of these, three encoded for cellulose synthase-like protein (LOCUS_008497-RA, LOCUS_031375-RA, and LOCUS_027455-RA) and a putative cellulose synthase A catalytic subunit (LOCUS_001677-RA) (FDR < 0.05; see Table S12 for complete list of genes). Additionally, Dolichyl-diphosphooligosaccharide-protein glycosyltransferase subunit (LOCUS_011739-RA) was found to be commonly and significantly induced across the varieties. A different gene (LOCUS_003933-RA) encoding for a similar peptide was also detected in the PDE model. These findings strongly suggest its participation in fiber polymerization. Its exact involvement in fiber synthesis will be an interesting area of study.

**Figure 8:**
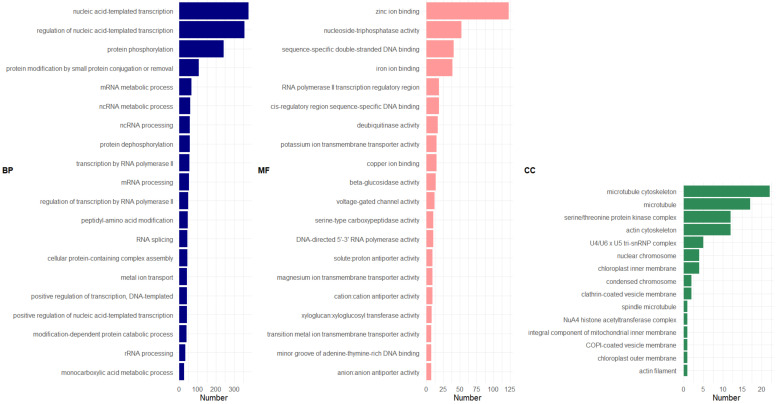
GO distribution of genes up-regulated in the abaca varieties using the NDE model for FQ across all abaca. Legend similar to Figure 6. Figure generated using ggplot2 [57] in Rstudio [21]. GO distribution analyzed using OmicsBox [35].

GO enrichment analysis revealed seven different transcript isoforms encoding for putative xyloglucan endotransglucosylase/hydrolase protein 28 associated with xyloglucan: xyloglucosyl transferase (MF; GO:0016762) (Figure 8) (see Figure S15, graphical representation of GO: MF) (also see Figures S16 and S17 for BP and CC graphical representations, respectively). Additionally, ß-glucosidase activity was found to be significantly enriched, which was consistent with the other models. Genes found to be associated with this GO term included ß-glucosidases and Glucan endo-1,3-ß-glucosidases (see Table S13 for a complete list of genes classified based on GO terms).

On the other hand, there appeared to be a consistent enrichment of cytoskeletons, microtubules, and actins (CC) across all models tested, largely signifying their participation during fiber synthesis. Such findings align with recent reports in the literature (e.g., [55,56]).

KEGG pathway analysis of NDE genes commonly and significantly expressed showed enrichment of enzymes associated with secondary metabolite biosynthesis. Two of these were (i) caffeoyl shikimate esterase (LOCUS_014428-RA), an enzyme central to the lignin biosynthetic pathway [58], and (ii) 4-coumarate-CoA ligase (LOCUS_015917-RA), a lignin biosynthesis-related enzyme [59].

A previous study showed that abaca fiber consists of 87.0%, 95.0%, and 7.8% glucose in the cell wall, glucan–lignin, and xylan–lignin (XL) fractions, respectively [60,61]. These findings corroborate those of the current study on the presence of genes encoding for enzymes involved in the synthesis of glucose, xylose, and lignin in abaca fiber.

#### 3.2.4. GDE between Resistant and Susceptible Varieties

We wanted to test genes differentially expressed between AbBTV-resistant (R) and -susceptible groups (S) (Table 2). We, therefore, implemented GDE between these two contrasting groups. Here, Abuab, Inosa and Tangongon were grouped as S, and Pacol and the backcrosses as the R group. This aimed to identify putative disease resistance genes. However, because these genotypes were not infected with the pathogen, the association of any GDE genes with resistance has yet to be concluded and would be an interesting subject in succeeding investigations.

The backcrosses, BC_2_ and BC_3_, harbor Pacol-specific alleles that did not map in the initial alignment against the Abuab reference. These unmapped reads were re-aligned against the *M. balbisiana* DH–PKW genome assembly (see Section 2). MA plot between the R and S groups showed a symmetrical datapoint indicating that normalization was effective (see Figure S18).

Results showed that 1126 (or 8.11%) of the 13,887 transcript isoforms were differentially expressed (FDR < 0.05), 510 of which were up-regulated in the R group (see Table S14 for list of genes differentially expressed between R and S). Three unique transcript orthologs that participate in disease resistance were found up-regulated in the R group (FDR < 0.05). These included Pathogenesis-related protein PR-1 (Mba05_g23810.1), which is involved in defense response [62]; CC-NBS-LRR (Mba01_g04170.1), for disease resistance, and Putative leaf rust 10 disease-resistance locus receptor-like protein kinase (Mba06_g10570.1). (Note: because Pacol is the resistant variety, we used the Mba* naming system of the *M. balbisiana* DH–PKW genome reference to which Pacol and Pacol-specific reads of the backcrosses were aligned.).

**Figure 9:**
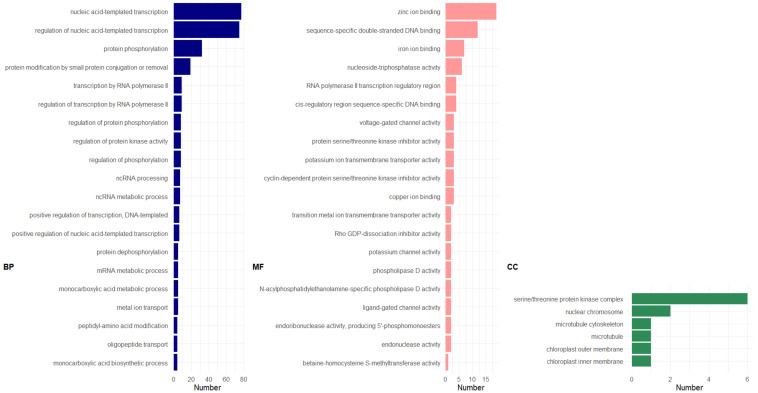
GO distribution of genes up-regulated in the abaca varieties using the GDE model between R and S genotype groups. Legend similar to Figure 6. Figure generated using ggplot2 [57] in Rstudio [21]. GO distribution analyzed using OmicsBox [35].

GO analysis of genes up-regulated in the R group showed enrichment of genes associated with Rho GDP-dissociation inhibitor activity (MF; GO:0005094), which participates in phagocytosis (Figure 9). These genes include Mba11_g07850.1 and Mba05_g13750.1, both of which encode for Rho GDP-dissociation inhibitor 1 (see list of genes associated with each GO term in Table S15, and Figures S19–S21 for graphical representations of BP, MF, and CC, respectively).

KEGG pathway analysis of the up-regulated genes in the R group showed enzymes enriched in Plant–Pathogen Interactions. These included cyclic nucleotide gated channel (e.g., Mba08_g30140.1), calcium-dependent protein kinase (e.g., Mba01_g13260.1), and 3-ketoacyl-CoA synthase (e.g., Mba03_g16550.1).

To further identify the molecular underpinnings of resistance of abaca against AbBTV, we recommend challenging these genotypes with the virus. Such an approach will elicit a transcriptional response of genes associated with the trait of interest. This is an interesting area for further inquiry.

## 4. Conclusions

A range of transcript orthologs underlying high fiber quality in abaca were identified following best practices in RNA-seq analysis. GO enrichment analysis showed several genes associated with polysaccharide synthesis including cellulose, callose, glucan, tubulins, actins, etc. KEGG pathway analysis also showed several genes encoding for enzymes involved in lignin biosynthesis. Their participation in fiber synthesis would be an interesting area for succeeding studies. Genes associated with disease resistance were identified, albeit modestly. These included Pathogenesis-related protein PR-1, CC-NBS-LRR, and Putative leaf rust 10 disease-resistance locus receptor-like protein kinase. KEGG pathway analysis further showed genes encoding for enzymes involved in Plant–Pathogen Interactions. The contribution of these candidate genes to fiber quality and pathogen resistance is something worth following up in future investigations.

## Figures and Tables

**Table 1:** Quality information and pre-library preparation quality assessment of the RNA samples.

Sample	Sample Quality Metrics			Pre-Library Prep Info		
	Conc. (ng/μL)	Volume (μL)	Total Amount (μg)	Conc. (ng/μL)	Conc. (nM)	Result
Abuab	462.418	21	9.711	25.1	105	Pass
BC_2_ (Bandala)	137.261	21	2.882	4.64	19.9	Pass
BC_3_	339.573	21	7.131	9.52	40.7	Pass
Inosa	150.325	21	3.157	2.65	10.9	Pass
Tangongon	366.006	21	7.686	7.07	29.8	Pass
Pacol	577.261	21	12.122	11.8	51.3	Pass

**Table 3:** Raw data information on the PE reads generated from sequencing of six *Musa* sp.

Sample	Total Read Bases (bp)	Total Reads	Q30 (%)
Abuab	3,487,334,262	34,528,062	95.28
BC_3_	4,327,484,582	42,846,382	95.49
BC_2_ (Bandala)	3,225,244,514	31,933,114	95.36
Inosa	4,690,066,098	46,436,298	95.3
Pacol	3,186,258,918	31,547,118	95.76
Tangongon	3,097,216,712	30,665,512	94.93

**Table 4:** Alignment rates of the reads generated from various *Musa* sp. varieties.

Variety	Uniquely Mapped Reads (%)	Reads Mapped to Multiple Loci (%)	Reads Unmapped (%)
Abuab	89.12	6.59	3.68
Tangongon	87.00	2.57	10.35
Inosa	83.11	3.43	13.35
BC_2_	59.96	3.03	35.87
BC_3_	65.37	2.66	31.40
Pacol	43.10	32.80	9.14

## Data Availability

All datasets have been uploaded in EMBL-EBI ArrayExpress (www.ebi.ac.uk/arrayexpress) with an assigned accession number: E-MTAB-10990.

## Supplementary Materials

The following supporting information can be downloaded at: https://www.mdpi.com/article/10.3390/genes13030519/s1, Supplementary Info S1. TapeStation profiles of the six *Musa* sp. before library preparation. Figure S1. Number of unique and common transcript orthologs between Abuab and DH–PKW reference assembly. A. Cluster counts of Abuab and DH–PKW. B. Common and unique cluster groups between the two reference assemblies. Figure S2. Mean (log ratio)–Average (mean average) or MA plot between Abuab and Pacol. Figure S3. Mean (log ratio)–Average (mean average) or MA plot between BC_3_ and Pacol. Figure S4. Mean (log ratio)–Average (mean average) or MA plot between BC_2_ (Bandala) and Pacol. Figure S5. Mean (log ratio)–Average (mean average) or MA plot between Inosa and Pacol. Figure S6. Mean (log ratio)–Average (mean average) or MA plot between Tangongon and Pacol. Figure S7. Graphical representations of gene ontology Biological Process enrichment (for PDE up-regulated genes). Figure S8. Graphical representations of gene ontology Molecular Function enrichment (for PDE up-regulated genes). Figure S9. Graphical representations of gene ontology Molecular Function enrichment (for PDE up-regulated genes). Figure S10. GO distribution for down-regulated transcript orthologs in abaca but up-regulated in Pacol using PDE model. Figure S11. MA plot between abaca and banana using GDE model. Figure S12. Graphical representation of GDE up-regulated, Biological Process. Figure S13. Graphical representation of GDE up-regulated, Molecular Function. Figure S14. Graphical representation of GDE up-regulated, Cellular Component. Figure S15. Graphical representation of NDE up-regulated, Molecular Function. Figure S16. Graphical representation of NDE up-regulated, Biological Process. Figure S17. Graphical representation of NDE up-regulated, Cellular Component. Figure S18. MA plot between R and S groups, GDE model. Figure S19. Graphical representation of BP for GDE R vs. S. Figure S20. Graphical representation MF, GDE R vs. S. Figure S21. Graphical representation CC, GDE R vs. S. Table S1. Complete list of genes differentially expressed between Abuab and Pacol using PDE model. Table S2. Complete list of genes differentially expressed between BC_3_ and Pacol using PDE model. Table S3. Complete list of genes differentially expressed between BC_2_ (Bandala) and Pacol using PDE model. Table S4. Complete list of genes differentially expressed between Inosa and Pacol using PDE model. Table S5. Complete list of genes differentially expressed between Tangongon and Pacol using PDE model. Table S6. Transcript orthologs commonly induced in the five abaca varieties which are down-regulated in Pacol. Table S7. List of genes classified based on GO enrichment analysis, up-regulated in PDE model. Table S8. GO analysis of genes down-regulated in abaca, PDE model. Table S9. GDE genes between abaca and Pacol using GDE for FQ. Table S10. GO analysis of genes up-regulated in abaca GDE model for FQ. Table S11. NDE among abaca varieties. Table S12. List of genes commonly up-regulated in abaca using NDE model. Table S13. GO analysis: list of genes significantly enriched in each GO term using NDE model. Table S14. List of significantly differentially expressed genes in the GDE model, R vs. S. Table S15. List of genes associated in each GO term for GDE model, R vs. S.

## References

[1] PHILFIDA Philippine Fiber Industry Development Authority. http://www.philfida.da.gov.ph/index.php/archived-articles/19-philippine-abaca-helps-in-global-environment-conservation.

[2] Ocampo K.R. Demand Rises for Philippine Abaca as Raw Material for PPE. Inquirer. https://newsinfo.inquirer.net/.

[3] Delicano J.A. (2018). A review on abaca fiber reinforced composites. Compos. Interfaces.

[4] Barba B.J., Madrid J.F., Penaloza D.P. (2020). A review of abaca fiber-reinforced polymer composites: Different modes of preparation and their applications. J. Chil. Chem. Soc..

[5] GBIF Global Biodiversity Information Facility. https://www.gbif.org/species/113660435.

[6] Halos S.C. (2008). The Abaca.

[7] D’Hont A., Denoeud F., Aury J.M., Baurens F.-C., Carreel F., Garsmeur O., Noel B., Bocs S., Droc G., Rouard M. (2012). The banana (*Musa acuminata*) genome and the evolution of monocotyledonous plants. Nature.

[8] Suthanthiram B., Uma S., Saraswathi M., Saravanakumar A., Arumugam C. (2015). Transcriptome analysis of banana (*Musa balbisiana*) based on next-generation sequencing technology. Turk. J. Agric. For..

[9] Galvez L.C., Koh R.B.L., Barbosa C.F.C., Asunto J.C., Catalla J.L., Atienza R.G., Costales K.T., Aquino V.M., Zhang D. (2021). Sequencing and de Novo Assembly of Abaca (*Musa textilis* Née) var. Abuab Genome. Genes.

[10] Spencer J.E. (1951). Abaca and the Philippines. Econ. Geogr..

[11] Lasalita-Zapico F.C., Aguilar C.H.M., Aujero J.M. (2010). Phenotypic variability of Manila hemp (*Musa textilis* L. Nee) genotypes in southern Mindanao Island, Philippines. J. Trop. Agric..

[12] Bhattacharyya D., Subasinghe A., Kim N.K., Friedrich K., Breuer U. (2015). Chapter 4—Natural fibers: Their composites and flammability characterizations. Multifunctionality of Polymer Composites.

[13] Armecin R., Gabon F. (2008). Biomass, organic carbon and mineral matter contents of abaca (*Musa textilis* Nee) at different stages of growth. Ind. Crop. Prod..

[14] Kumar P.L., Selvarajan R., Iskra-Caruana M.L., Chabannes M., Hanna R. (2014). Biology, Etiology, and Control of Virus Diseases of Banana and Plantain. Adv. Virus Res..

[15] Lalusin A.G., Villavicencio M.L.H. (2015). Abaca (*Musa textilis* Nee) Breeding in the Philippines. Industrial Crops; Breeding for Bioenergy and Bioproducts.

[16] (2017). PHILFIDA. http://www.philfida.da.gov.ph/index.php/news-articles/116-3-abaca-varities-now-regsitered-with-nsic.

[17] Biswas S., Agrawal Y.N., Mucyn T.S., Dang J.L., Jones C.D. (2013). Biological Averaging in RNA-Seq. arXiv.

[18] Assefa A.T., Vandesompele J., Thas O. (2020). On the utility of RNA sample pooling to optimize cost and statistical power in RNA sequencing experiments. BMC Genom..

[19] Miao G., Qin Y., Guo J., Zhang Q., Bao Y. (2021). Transcriptome characterization and expression profile of *Coix lacryma-jobi* L. in response to drought. PLoS ONE.

[20] Yang X., Zhao T., Rao P., Gao K., Yang X., Chen Z., An X. (2019). Transcriptome profiling of Populus tomentosa under cold stress. Ind. Crop. Prod..

[21] RStudio Team (2020). RStudio: Integrated Development for R.

[22] Andrews S. (2010). FastQC: A Quality Control Tool for High Throughput Sequence Data. http://www.bioinformatics.babraham.ac.uk/projects/fastqc.

[23] Dobin A., Davis C.A., Schlesinger F., Drenkow J., Zaleski C., Jha S., Batut P., Chaisson M., Gingeras T.R. (2013). STAR: Ultrafast universal RNA-seq aligner. Bioinformatics.

[24] Wang Z., Miao H., Liu J., Xu B., Yao X., Xu C., Zhao S., Fang X., Jia C., Wang J. (2019). *Musa balbisiana* genome reveals subgenome evolution and functional divergence. Nat. Plants.

[25] Li H., Handsaker B., Wysoker A., Fennell T., Ruan J., Homer N., Marth G., Abecasis G., Durbin R. (2009). 1000 Genome Project Data Processing Subgroup, the Sequence Alignment/Map format and SAMtools. Bioinformatics.

[26] Liao Y., Smyth G.K., Shi W. (2014). Feature Counts: An efficient general purpose program for assigning sequence reads to genomic features. Bioinformatics.

[27] Ballereau S., Couturier D.L., Dunning M., Edwards A., Sawle A. (2019). RNA-seq Analysis in R: Counting Reads with SubRead. https://bioinformatics-core-shared-training.github.io/.

[28] Hardcastle T.J. (2021). BaySeq: Empirical Bayesian Analysis of Patterns of Differential Expression in Count Data. https://bioconductor.org/packages/release/bioc/html/baySeq.html.

[29] Hardcastle T.J., Kelly K.A. (2010). BaySeq: Empirical Bayesian methods for identifying differential expression in sequence count data. BMC Bioinform..

[30] Xu L., Dong Z., Fang L., Luo Y., Wei Z., Guo H., Zhang G., Gu Y.Q., Coleman-Derr D., Xia Q. (2019). OrthoVenn2: A web server for whole-genome comparison and annotation of orthologous clusters across multiple species. Nucleic Acids Res..

[31] Robinson M.D., Oshlack A. (2010). A scaling normalization method for differential expression analysis of RNA-seq data. Genome Biol..

[32] Benjamini Y., Hochberg Y. (1995). Controlling the False Discovery Rate: A Practical and Powerful Approach to Multiple Testing. J. R. Stat. Soc. Ser. B Methodol..

[33] Bell G.D., Kane N.C., Rieseberg L.H., Adams K.L. (2013). RNA-Seq Analysis of Allele-Specific Expression, Hybrid Effects, and Regulatory Divergence in Hybrids Compared with Their Parents from Natural Populations. Genome Biol. Evol..

[34] Shi X., Ng D.W.-K., Zhang C., Comai L., Ye W., Chen Z.J. (2012). Cis- and trans-regulatory divergence between progenitor species determines gene-expression novelty in Arabidopsis allopolyploids. Nat. Commun..

[35] (2019). BioBam Bioinformatics. OmicsBox—Bioinformatics Made Easy (Version 2.0.29). https://www.biobam.com/omicsbox.

[36] Moriya Y., Itoh M., Okuda S., Yoshizawa A.C., Kanehisa M. (2007). KAAS: An automatic genome annotation and pathway reconstruction server. Nucleic Acids Res..

[37] Labrador D.A., Lalusin A.G., Mendoza M.R.R., dela Viña C.B. (2020). Morphological Characterization and Karyotype Analysis of Abaca (*Musa textilis* Nee) and Its Hybrids with *Musa balbisiana* Colla. Philipp. Agric. Sci..

[38] Parac E.P., Lalusin A.G., Pangga I.B., Sta Cruz F.C. (2020). Characteristics of Selected Hybrids of Abaca (*Musa textilis* Nee) with Resistance to Bunchy Top. Philipp. Agric. Sci..

[39] Boguero A.P.B., Parducho M.A.L., Mendoza M.R., Abustan M.A., Lalusin A.G. (2016). Molecular Screening of Abaca (*Musa textilis* Nee). Philipp. J. Crop Sci..

[40] CFC, UNIDO, FAO, FIDA (2004). Abaca Improvement of Fiber Extraction and Identification of Higher Yielding Varieties. Final Technical Report CFC/FIGHF/09. Activities in the Philippines. https://www.yumpu.com/en/document/view/27575439/abaca-activities-in-the-philippines-unido.

[41] Lalusin A.G. (2020). Revitalizing the Abaca Industry through S & T Interventions for Higher Crop Productivity Using High-Yielding and Bunchy Top-Resistant Abaca Hybrids.

[42] Parducho M.A.L., Rama RA B., Lalusin A.G. (2020). Stability Analysis of BC_2_ Abaca (*Musa textilis* Nee) Hybrids across Different Locations in the Philippines. Philipp. J. Crop Sci. PJCS.

[43] Zhou Y., Zhu J., Tong T., Wang J., Lin B., Zhang J. (2019). A statistical normalization method and differential expression analysis for RNA-seq data between different species. BMC Bioinform..

[44] Galvez L.C., Catalla J.L., Borromeo T.H., Altoveros N.C. (2018). Abaca Germplasm Conservation.

[45] Bao Y., Hu G., Grover C.E., Conover J., Yuan D., Wendel J.F. (2019). Unraveling cis and trans regulatory evolution during cotton domestication. Nat. Commun..

[46] Lovell J.T., Schwartz S., Lowry D.B., Shakirov E.V., Bonnette J.E., Weng X., Wang M., Johnson J., Sreedasyam A., Plott C. (2016). Drought responsive gene expression regulatory divergence between upland and lowland ecotypes of a perennial C4 grass. Genome Res..

[47] Ereful N.C., Laurena A., Liu L.-Y., Kao S.-M., Tsai E., Greenland A., Powell W., Mackay I., Leung H. (2021). Unraveling regulatory divergence, heterotic malleability, and allelic imbalance switching in rice due to drought stress. Sci. Rep..

[48] Saragih S.W., Wirjosentono B., Eddyanto M.Y. (2019). Extraction and Characterization of Cellulose from Abaca Pseudo Stem (*Musa textilis*). J. Phys. Conf. Ser..

[49] Sinha A.K., Bhattacharya S., Narang H.K. (2021). Abaca fibre reinforced polymer composites: A review. J. Mater. Sci..

[50] Uniprot. https://www.uniprot.org/.

[51] Wang G.F., He Y., Strauch R., Olukolu B.A., Nielsen D., Li X., Balint-Kurti P.J. (2015). Maize Homologs of Hydroxycinnamoyltransferase, a Key Enzyme in Lignin Biosynthesis, Bind the Nucleotide Binding Leucine-Rich Repeat Rp1 Proteins to Modulate the Defense Response. Plant Physiol..

[52] Serrani-Yarce J.C., Escamilla-Trevino L., Barros J., Gallego-Giraldo L., Pu Y., Ragauskas A., Dixon R.A. (2021). Targeting hydroxycinnamoyl CoA: Shikimate hydroxycinnamoyl transferase for lignin modification in *Brachypodium distachyon*. Biotechnol. Biofuels.

[53] Heberle H., Meirelles G.V., Da Silva F.R., Telles G.P., Minghim R. (2015). InteractiVenn: A web-based tool for the analysis of sets through Venn diagrams. BMC Bioinform..

[54] Li S., Sun T., Ren H. (2015). The functions of the cytoskeleton and associated proteins during mitosis and cytokinesis in plant cells. Front. Plant Sci..

[55] Chen B., Zhao J., Fu G., Pei X., Pan Z., Li H., Ahmed H., He S., DU X. (2021). Identification and expression analysis of Tubulin gene family in upland cotton. J. Cotton Res..

[56] Pydiura N., Pirko Y., Galinousky D., Postovoitova A., Yemets A., Kilchevsky A., Blume Y. (2019). Genome-wide identification. Phylogenetic classification. And exon-intron structure characterization of the tubulin and actin genes in flax (*Linum usitatissimum*). Cell Biol. Int..

[57] Wickham H. (2016). Ggplot2: Elegant Graphics for Data Analysis.

[58] Vanholme R., Cesarino I., Rataj K. (2013). Caffeoyl Shikimate Esterase (CSE) Is an Enzyme in the Lignin Biosynthetic Pathway in *Arabidopsis*. Science.

[59] Liu Q., Luo L., Zheng L. (2018). Lignins: Biosynthesis and Biological Functions in Plants. Int. J. Mol. Sci..

[60] Del Río J.C., Prinsen P., Cadena E.M., Martínez Á.T., Gutiérrez A., Rencoret J. (2016). Lignin-carbohydrate complexes from sisal (*Agave sisalana*) and abaca (*Musa textilis*): Chemical composition and structural modifications during the isolation process. Planta.

[61] Del Río J.C., Gutiérrez A. (2006). Chemical composition of abaca (*Musa textilis*) leaf fibers used for manufacturing of high quality paper pulps. J. Agric. Food. Chem..

[62] Breen S., Williams S.J., Outram M., Kobe B., Solomon P.S. (2017). Emerging Insights into the Functions of Pathogenesis-Related Protein 1. Trends Plant Sci..

