# Supplementary material for: RNA-Seq Reveals Differentially Expressed Genes Associated with High Fiber Quality in Abaca (Musa textilis Nee)
Source: Genes (Basel). 2022 Mar 15;13(3):519. doi: 10.3390/genes13030519 (PMC8953247; doi:10.3390/genes13030519)
Supplement: Supplementary file 1 [file genes-13-00519-s001.zip › genes-1568159-supplementary/Supplementary Materials Final/Supplementary Info S1. Tapestation profiles of the six Musa sp.2.pdf]

Supplementary Info 1. Tapestation profiles of the six *Musa* sp.

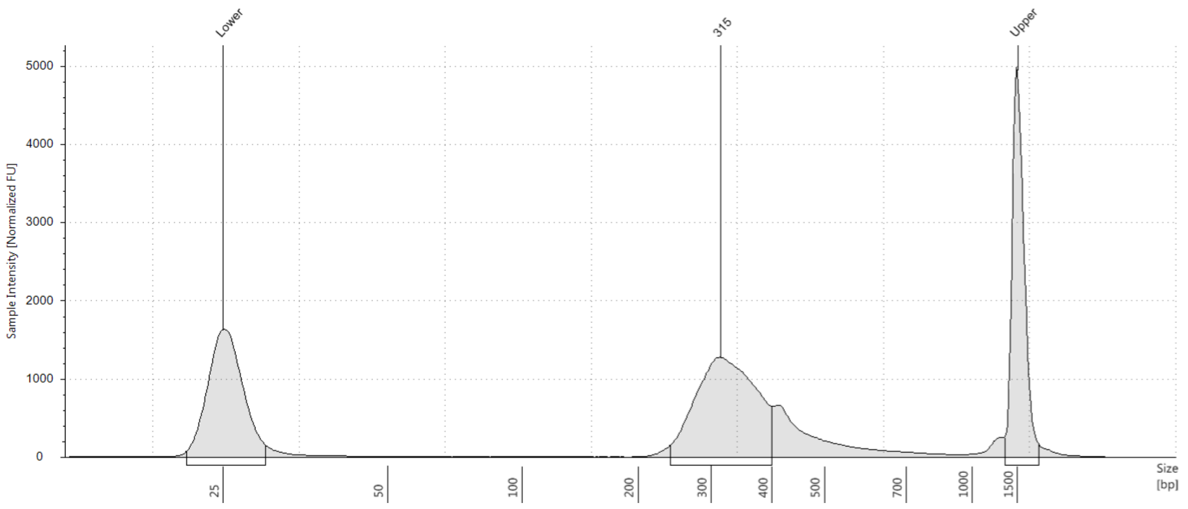


A. Pacol

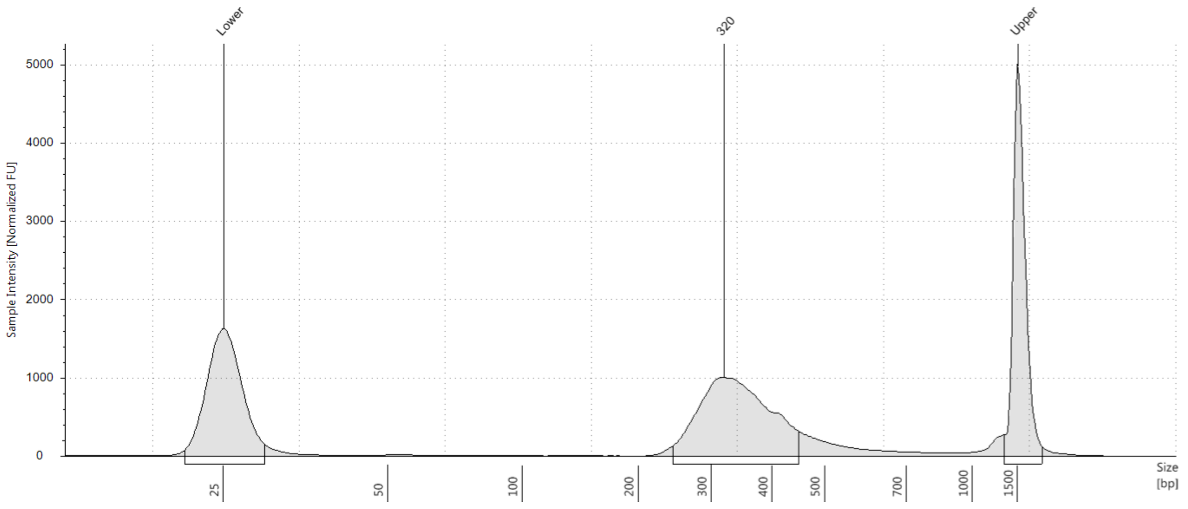


B. BC3

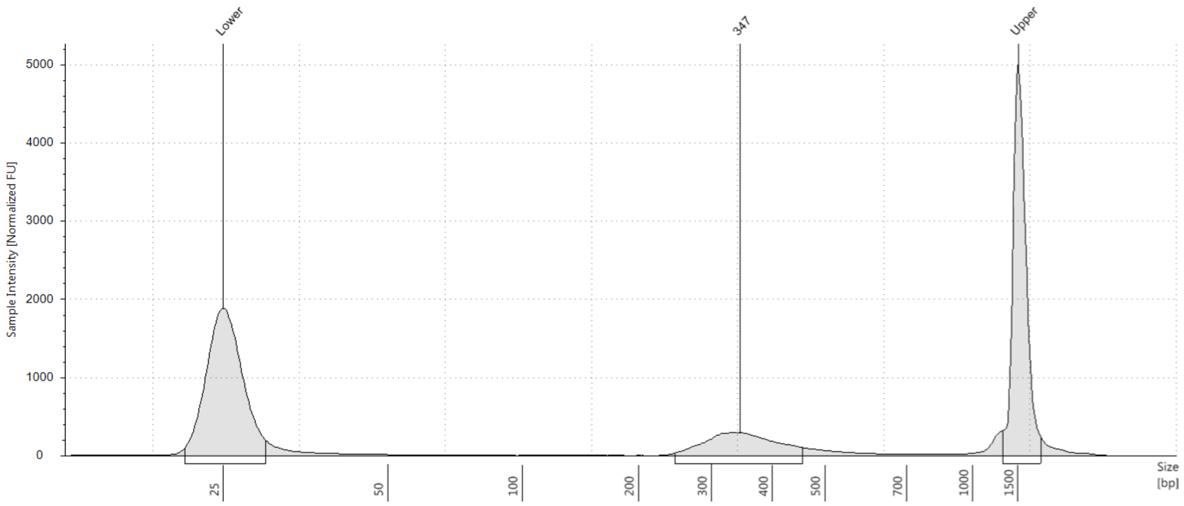


C. Inosa

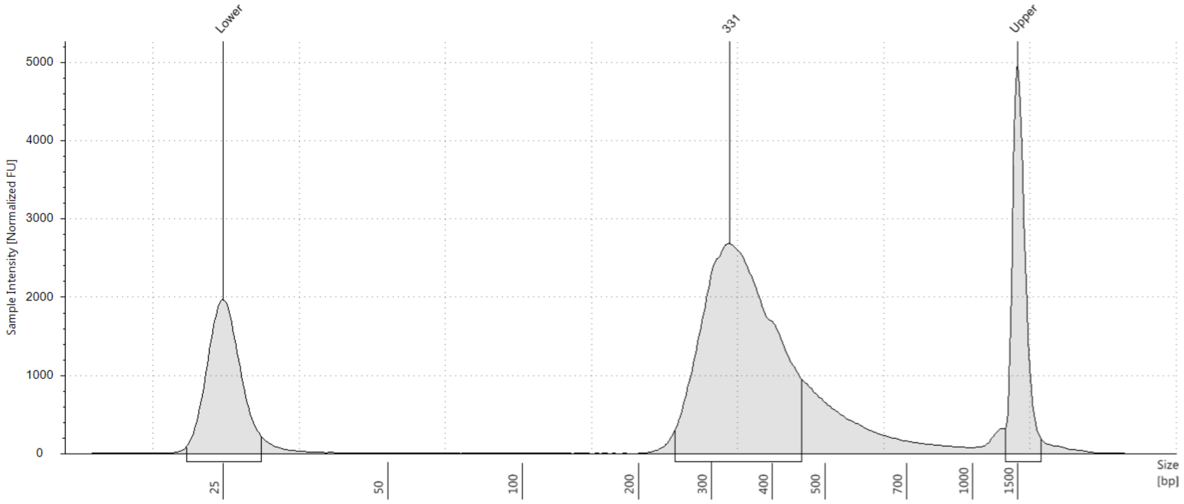


D. Abuab

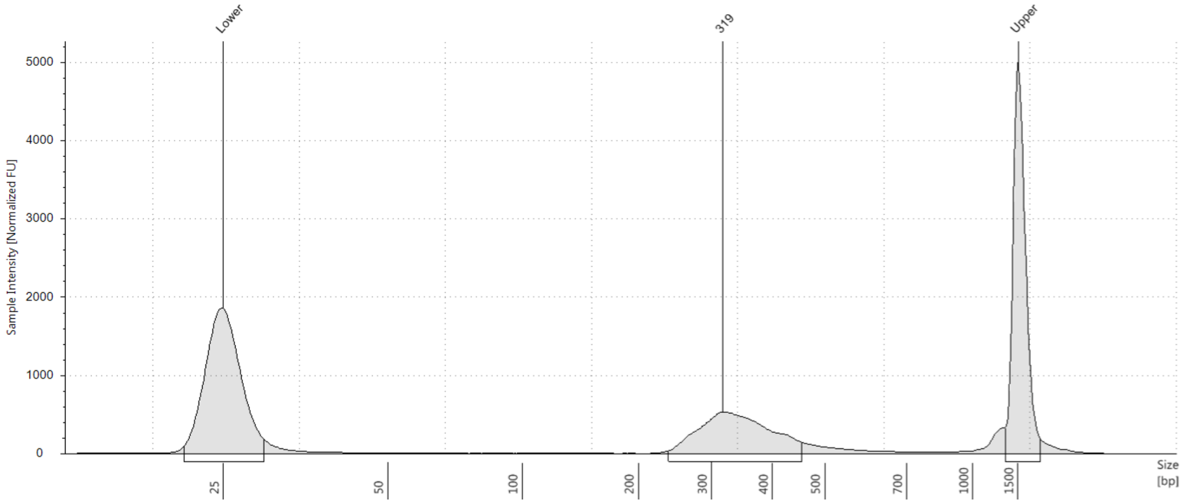


E. Bandala (BC2)

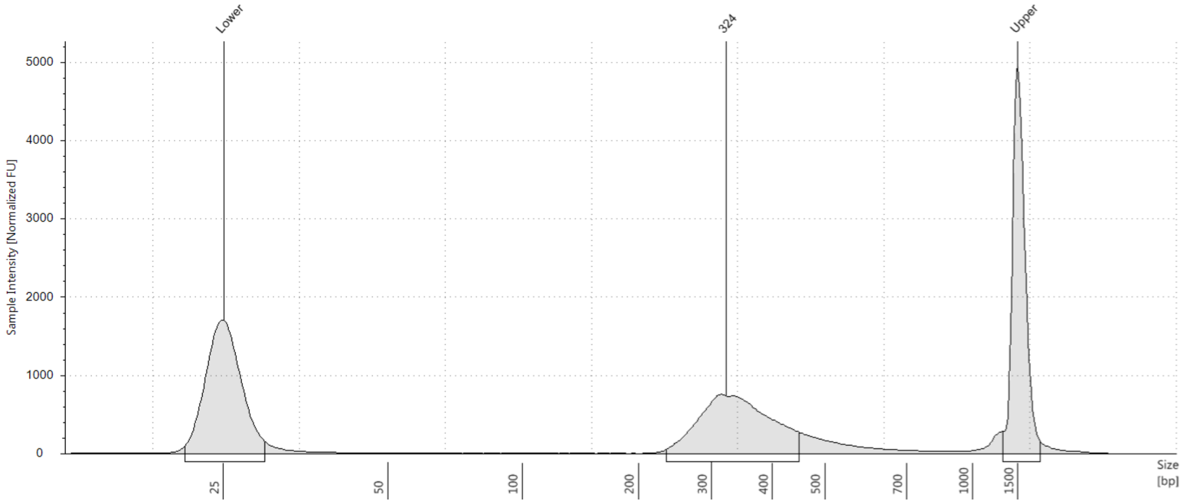


F. Tangongon
