# Supplementary figures and images for: RNA-Seq Reveals Differentially Expressed Genes Associated with High Fiber Quality in Abaca (Musa textilis Nee)
Source: Genes (Basel). 2022 Mar 15;13(3):519. doi: 10.3390/genes13030519 (PMC8953247; doi:10.3390/genes13030519)

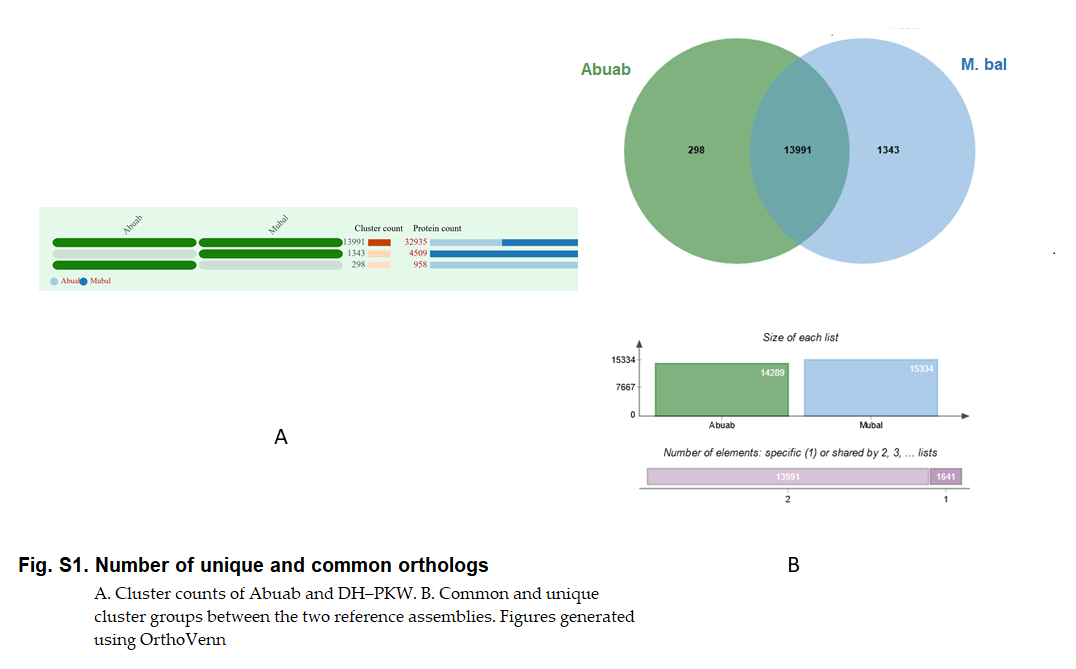

Supplement: Supplementary file 1 [file genes-13-00519-s001.zip › genes-1568159-supplementary/Supplementary Materials Final/Figure S1. Number of unique and common orthologs.png]

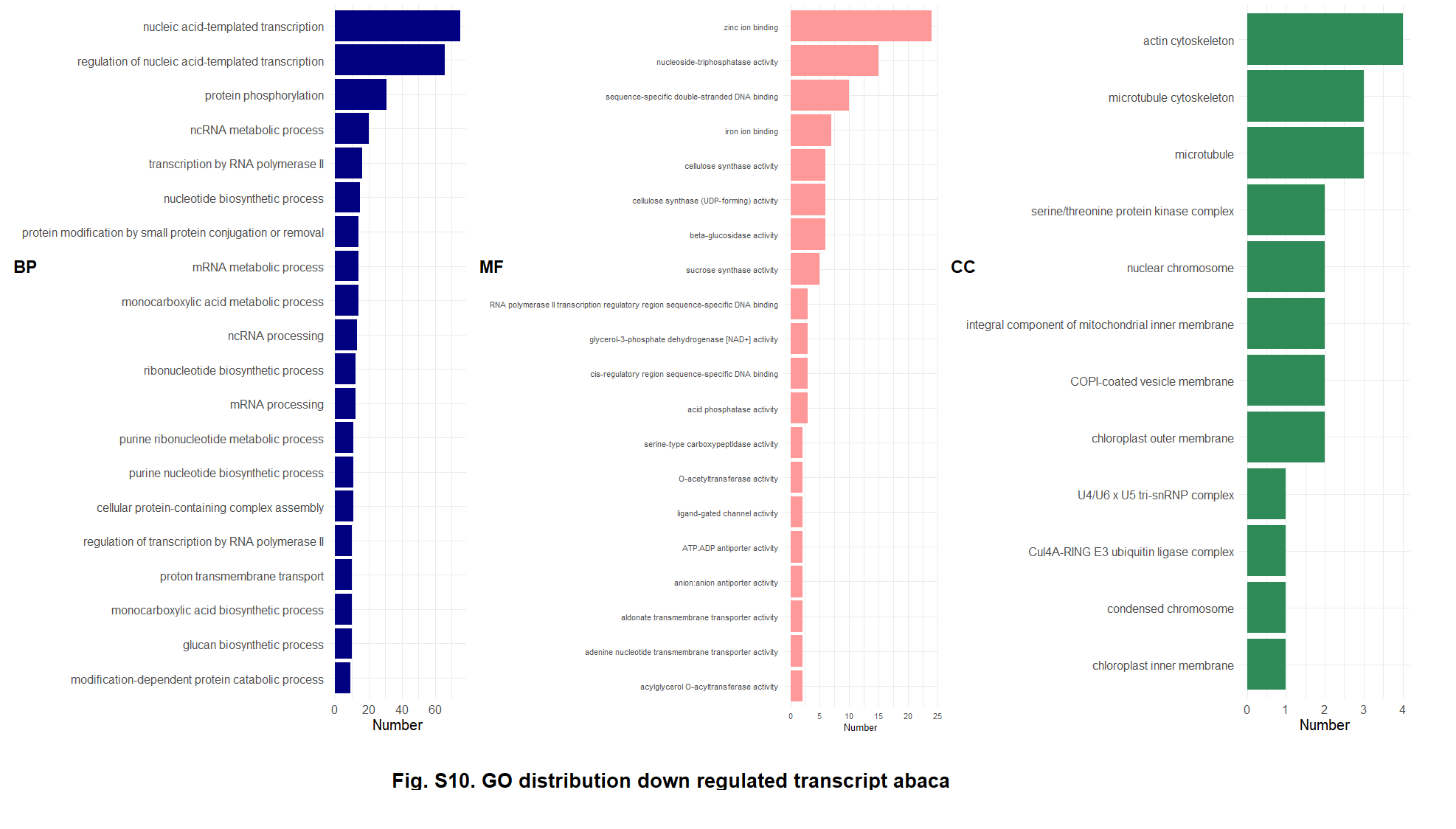

Supplement: Supplementary file 1 [file genes-13-00519-s001.zip › genes-1568159-supplementary/Supplementary Materials Final/Figure S10. GO distribution down regulated transcript abaca.png]

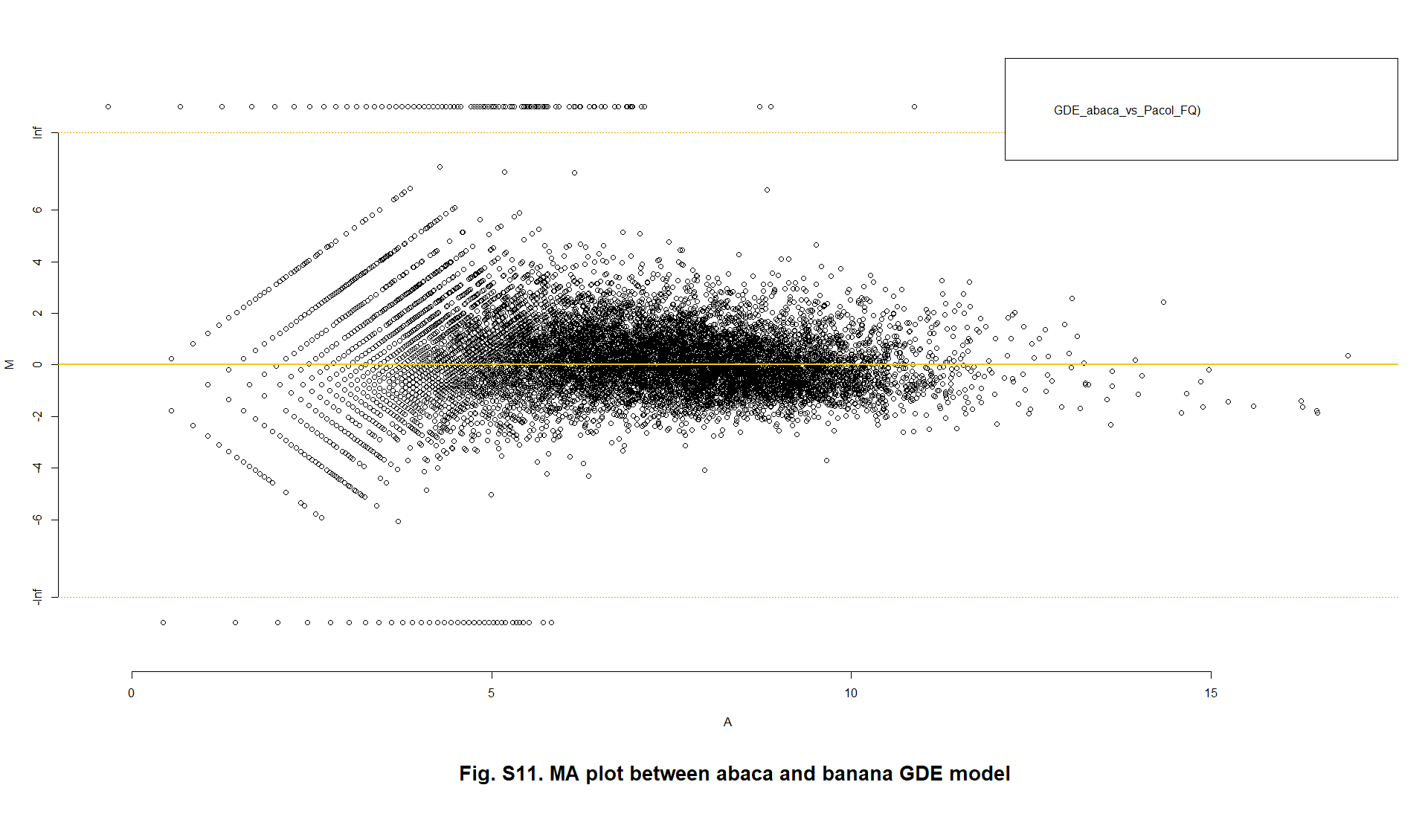

Supplement: Supplementary file 1 [file genes-13-00519-s001.zip › genes-1568159-supplementary/Supplementary Materials Final/Figure S11. MA plot between abaca and banana GDE model.png]

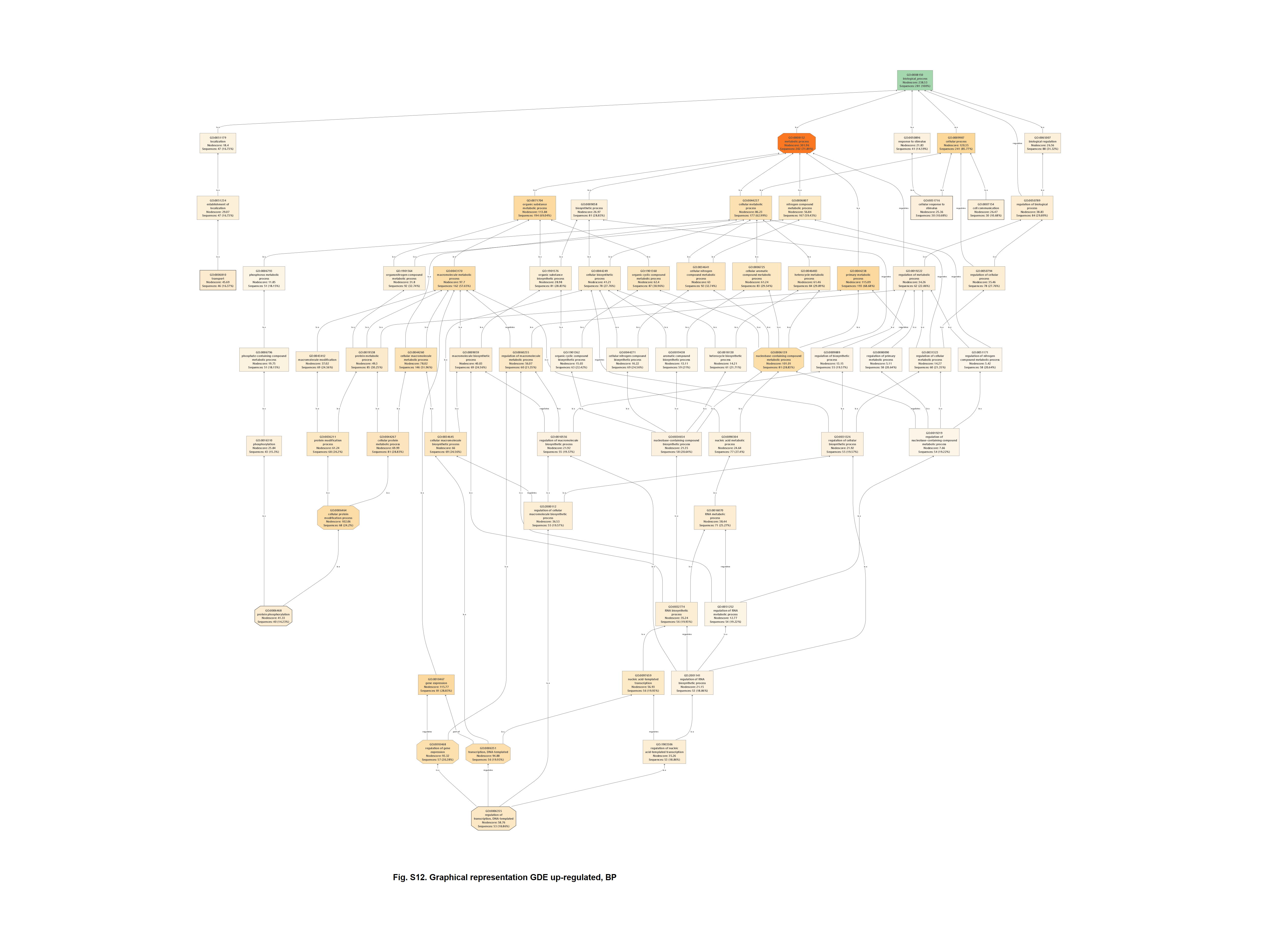

Supplement: Supplementary file 1 [file genes-13-00519-s001.zip › genes-1568159-supplementary/Supplementary Materials Final/Figure S12. Graphical representation GDE up-regulated, BP.png]

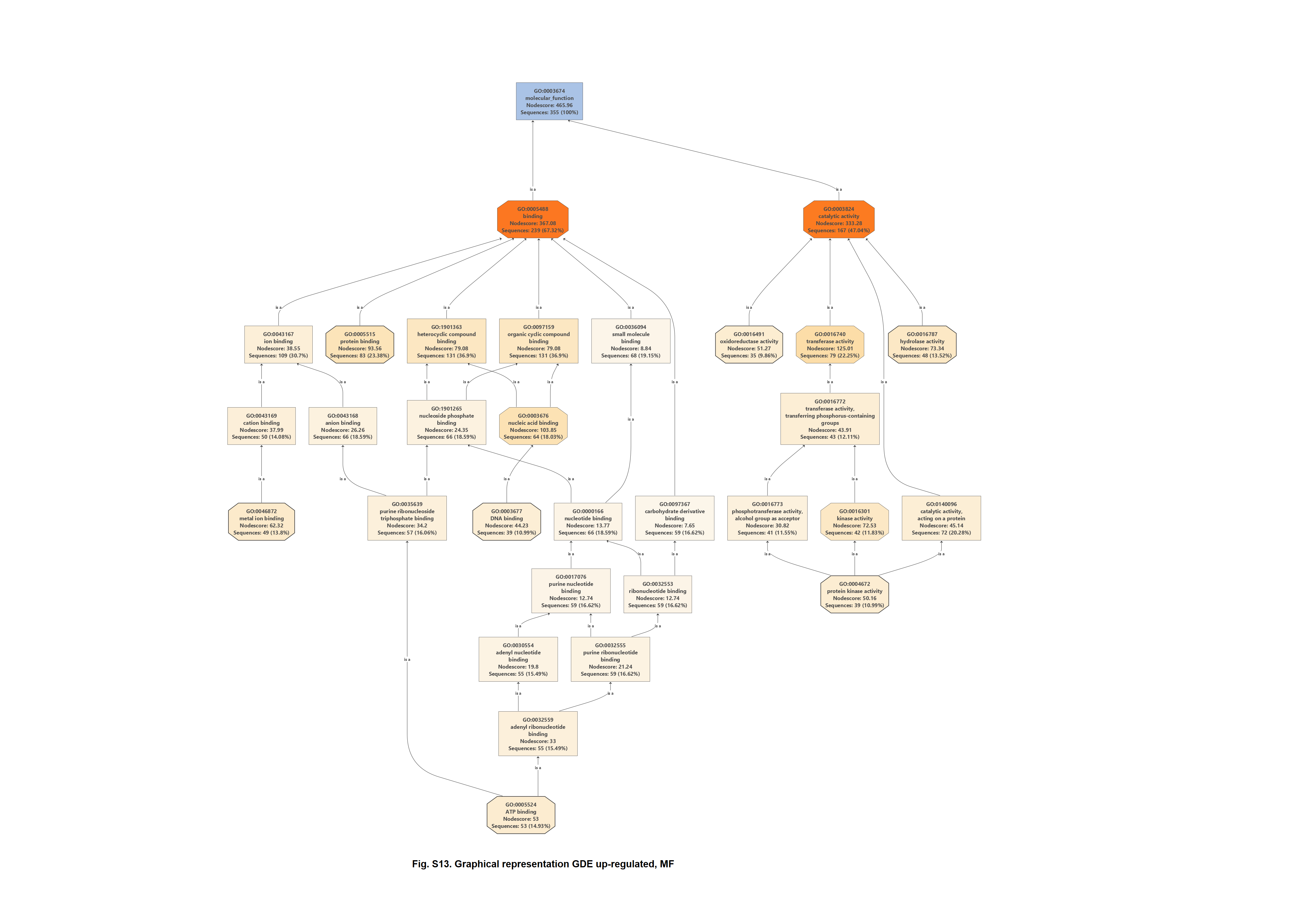

Supplement: Supplementary file 1 [file genes-13-00519-s001.zip › genes-1568159-supplementary/Supplementary Materials Final/Figure S13. Graphical representation GDE up-regulated, MF.png]

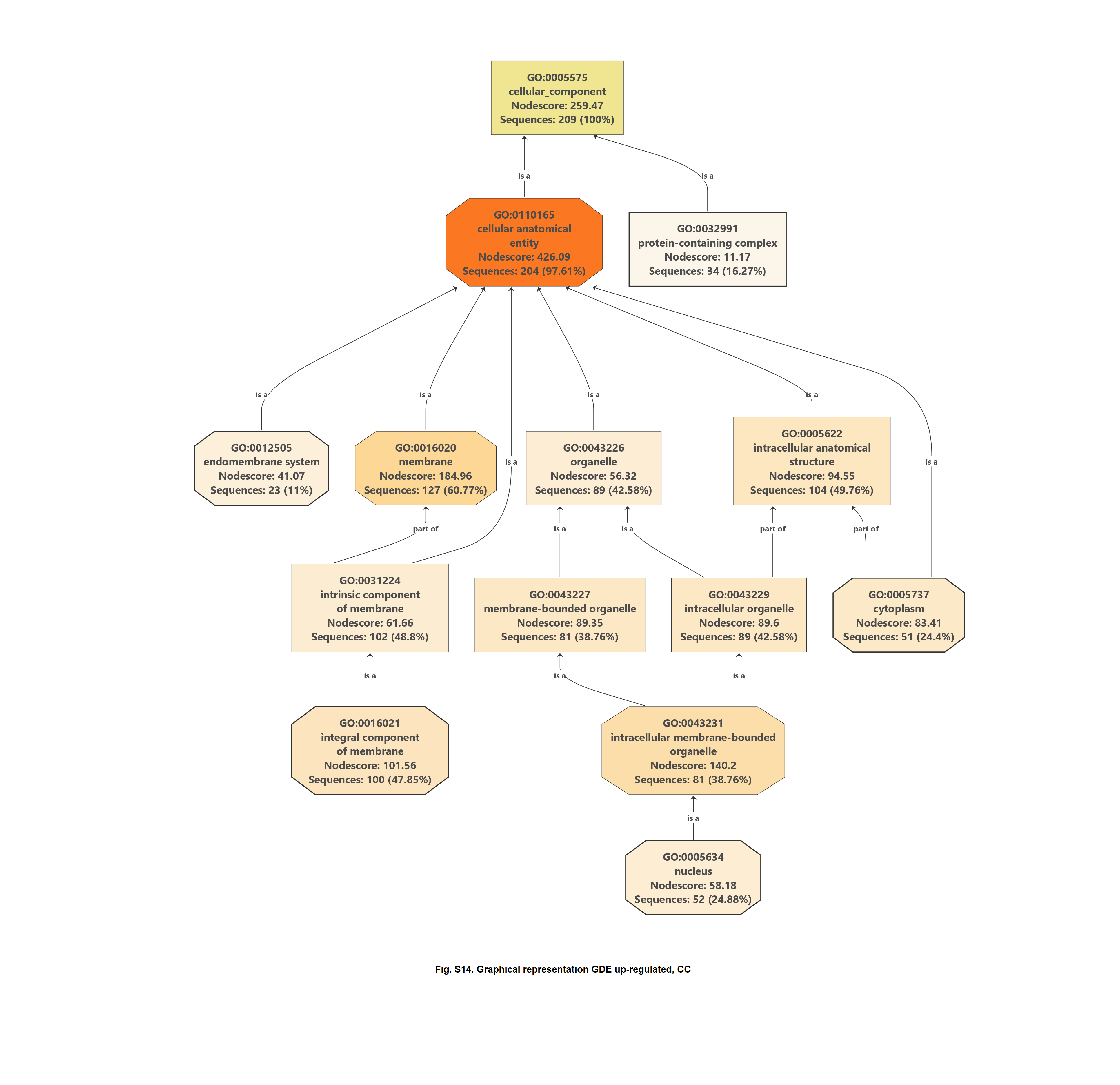

Supplement: Supplementary file 1 [file genes-13-00519-s001.zip › genes-1568159-supplementary/Supplementary Materials Final/Figure S14. Graphical representation GDE up-regulated, CC.png]

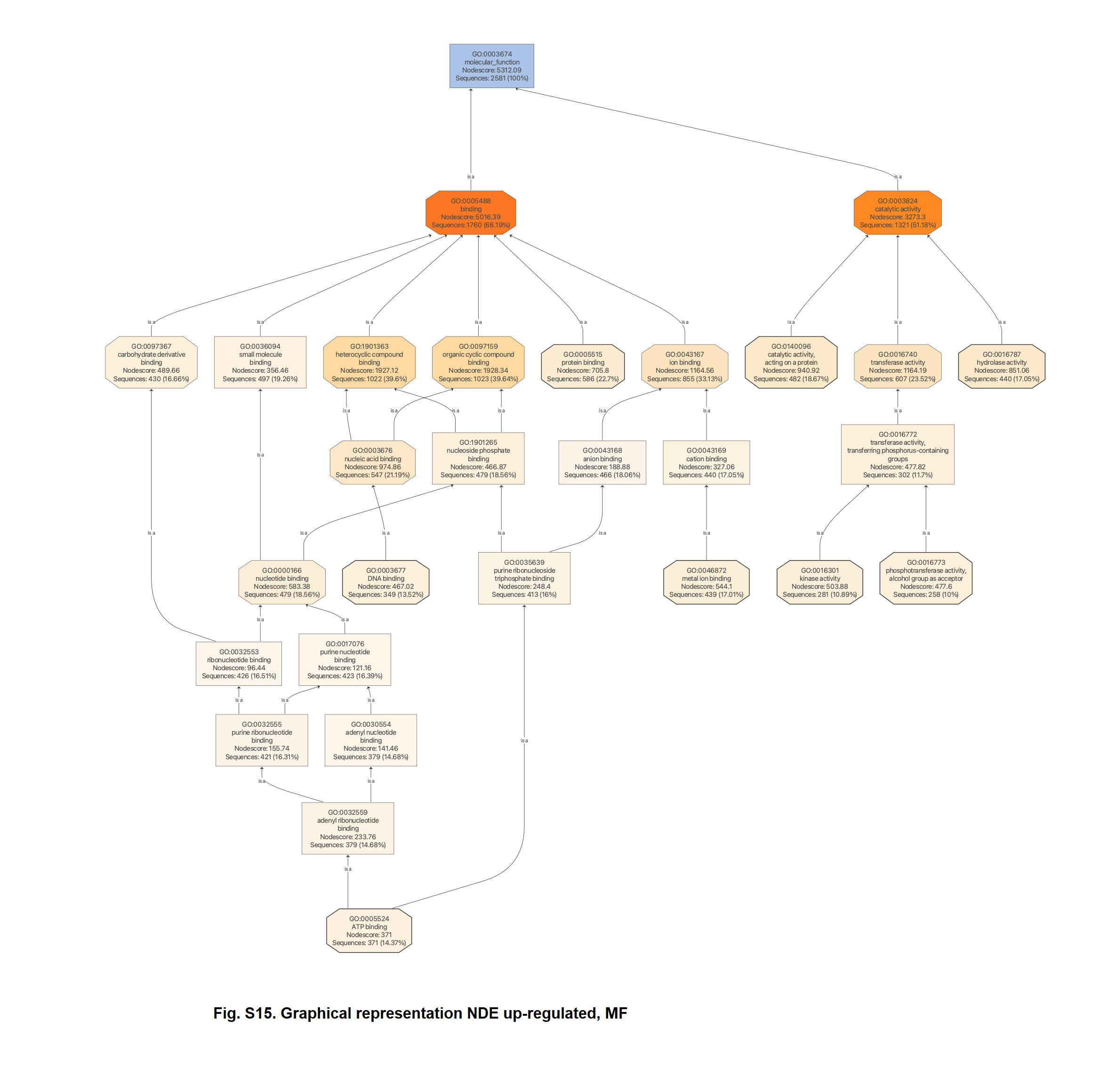

Supplement: Supplementary file 1 [file genes-13-00519-s001.zip › genes-1568159-supplementary/Supplementary Materials Final/Figure S15. Graphical representation NDE up-regulated, MF.png]

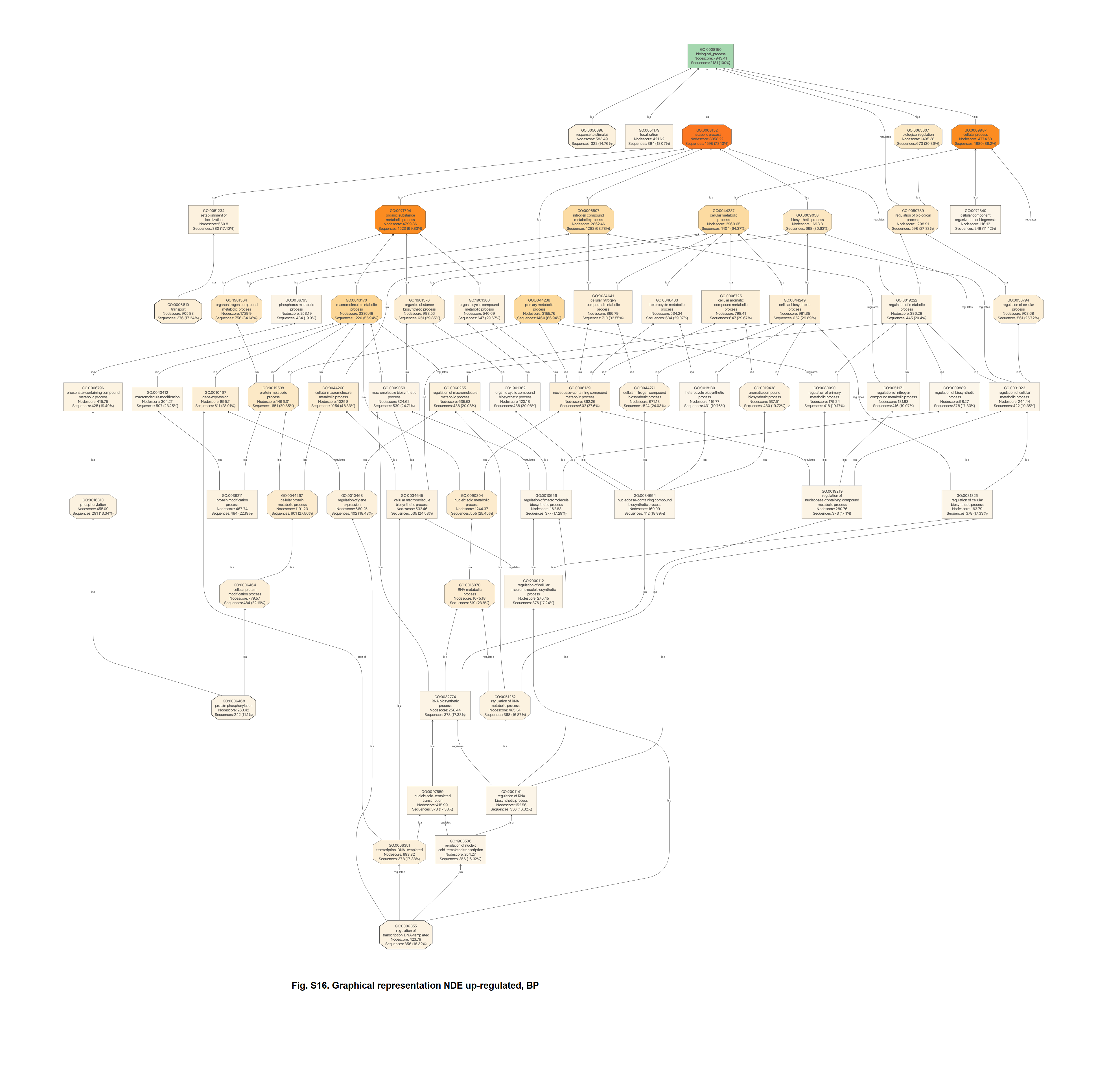

Supplement: Supplementary file 1 [file genes-13-00519-s001.zip › genes-1568159-supplementary/Supplementary Materials Final/Figure S16. Graphical representation NDE up-regulated, BP.png]

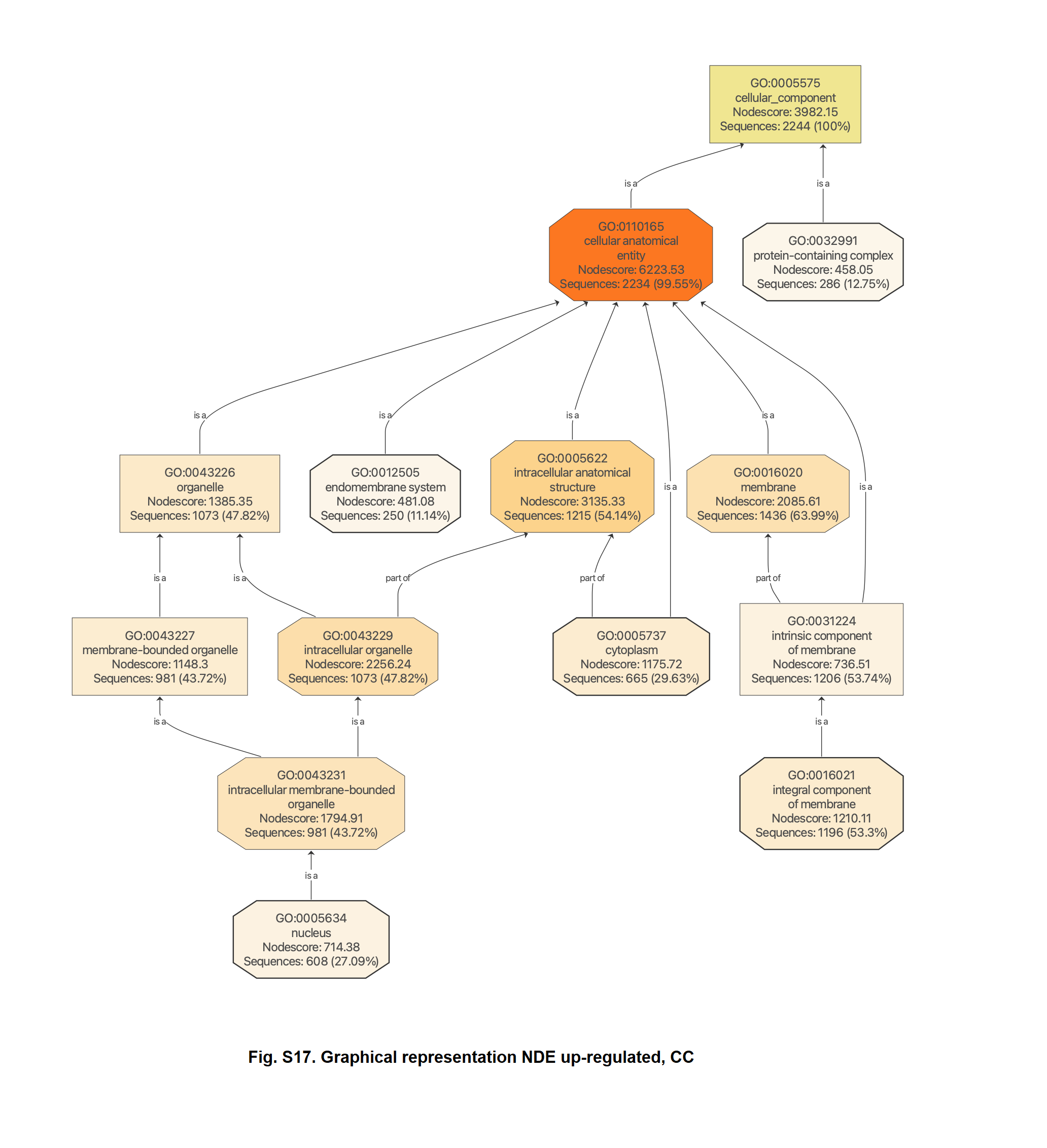

Supplement: Supplementary file 1 [file genes-13-00519-s001.zip › genes-1568159-supplementary/Supplementary Materials Final/Figure S17. Graphical representation NDE up-regulated, CC.png]

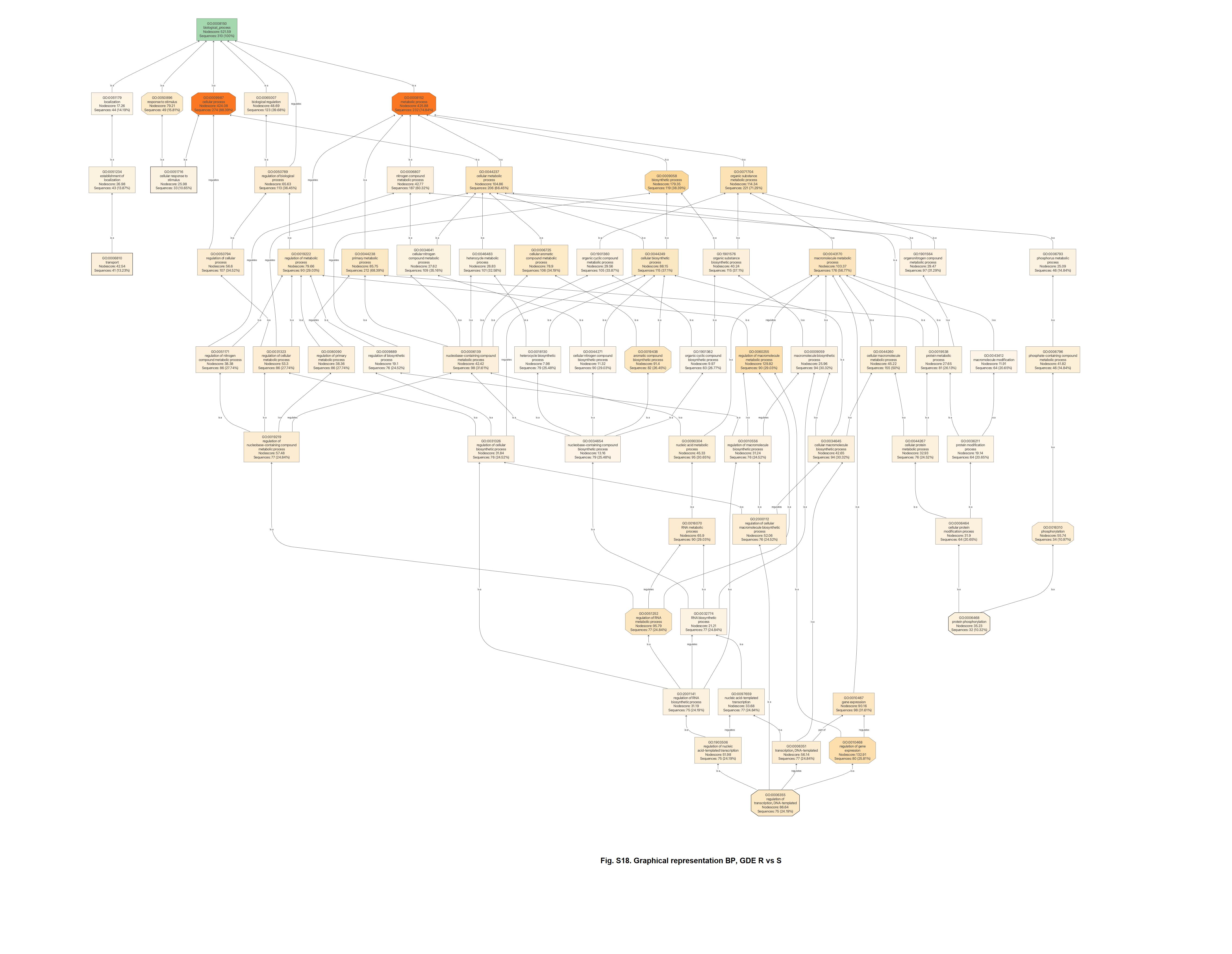

Supplement: Supplementary file 1 [file genes-13-00519-s001.zip › genes-1568159-supplementary/Supplementary Materials Final/Figure S18. Graphical representation BP, GDE R vs S.png]

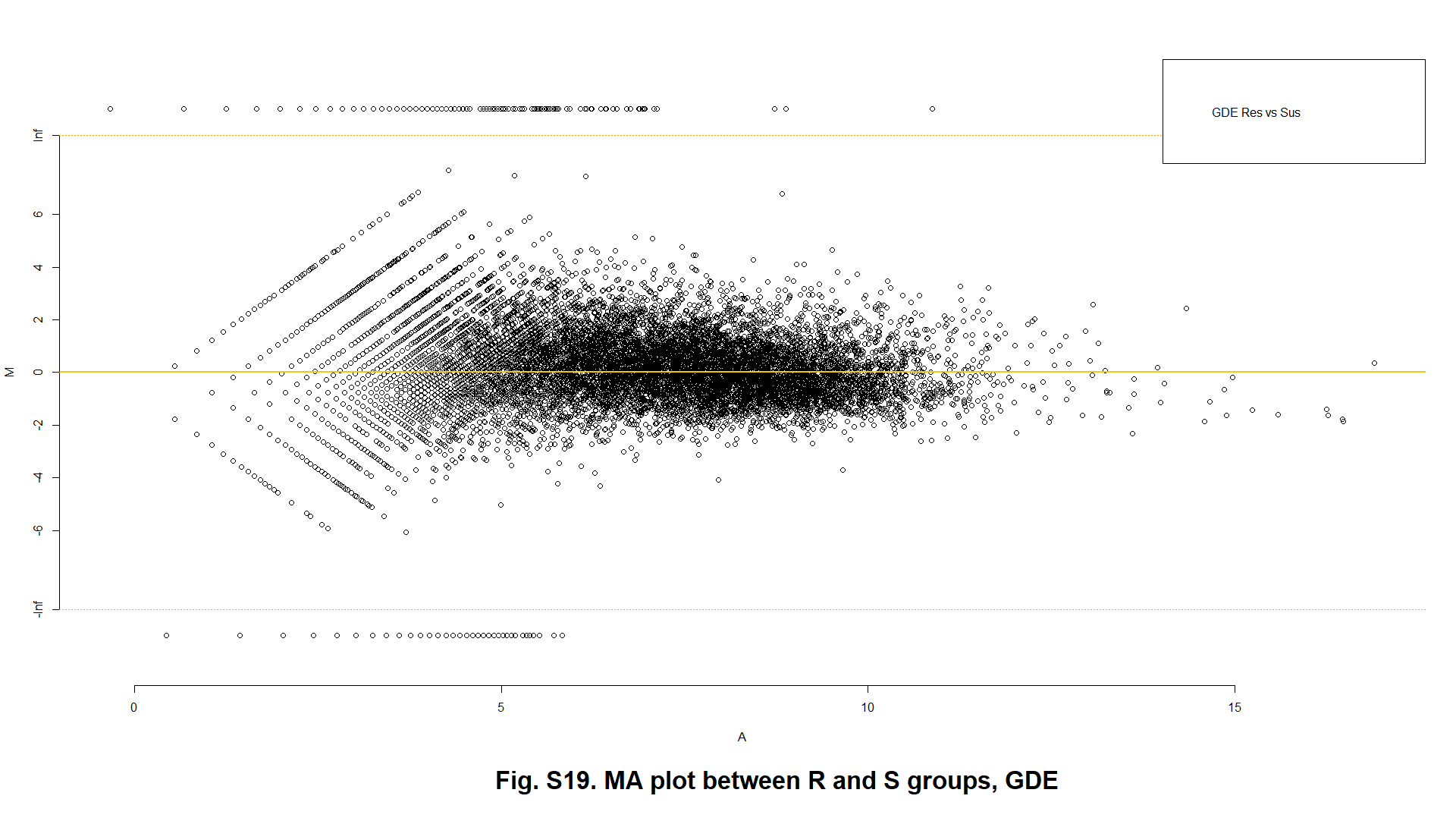

Supplement: Supplementary file 1 [file genes-13-00519-s001.zip › genes-1568159-supplementary/Supplementary Materials Final/Figure S19. MA plot between R and S groups, GDE.png]

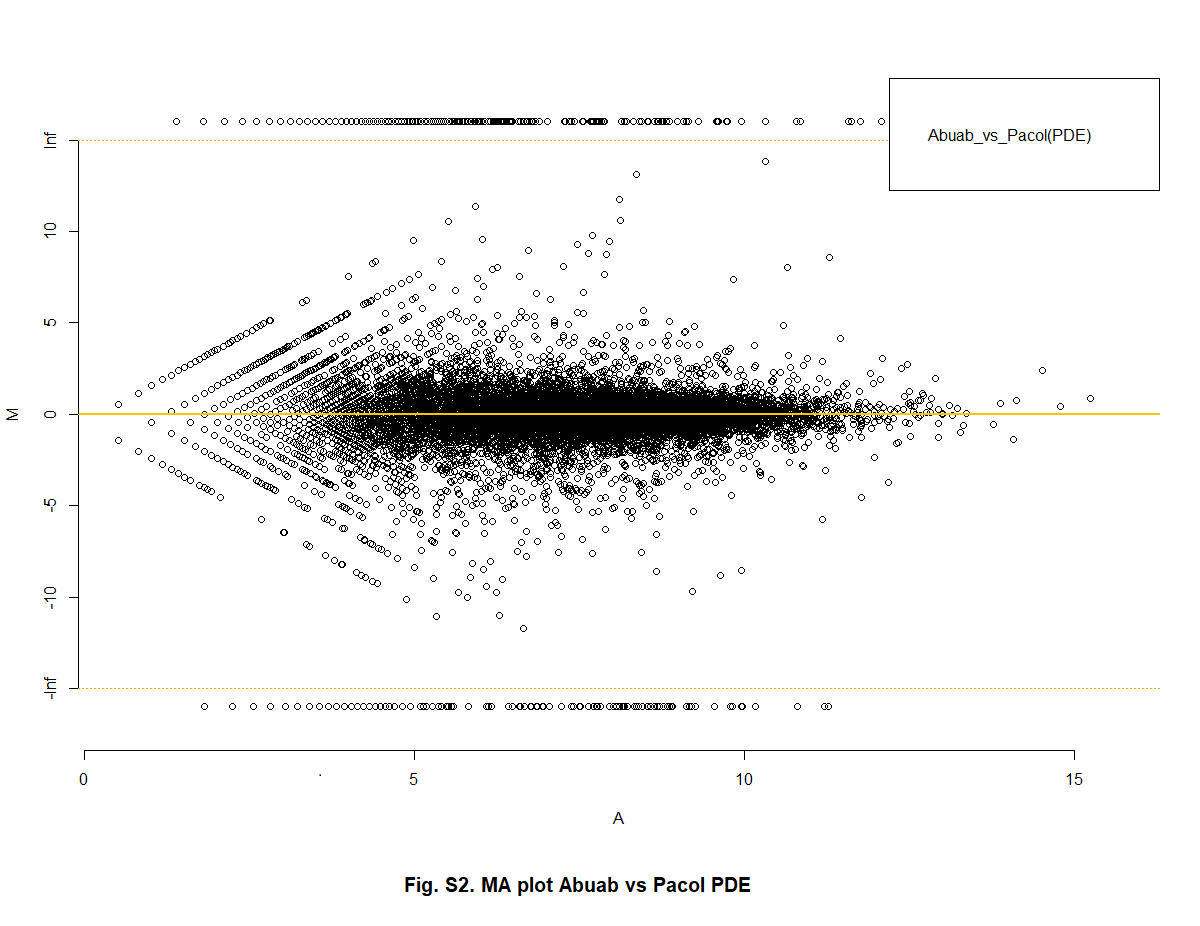

Supplement: Supplementary file 1 [file genes-13-00519-s001.zip › genes-1568159-supplementary/Supplementary Materials Final/Figure S2. MA plot Abuab vs Pacol PDE.png]

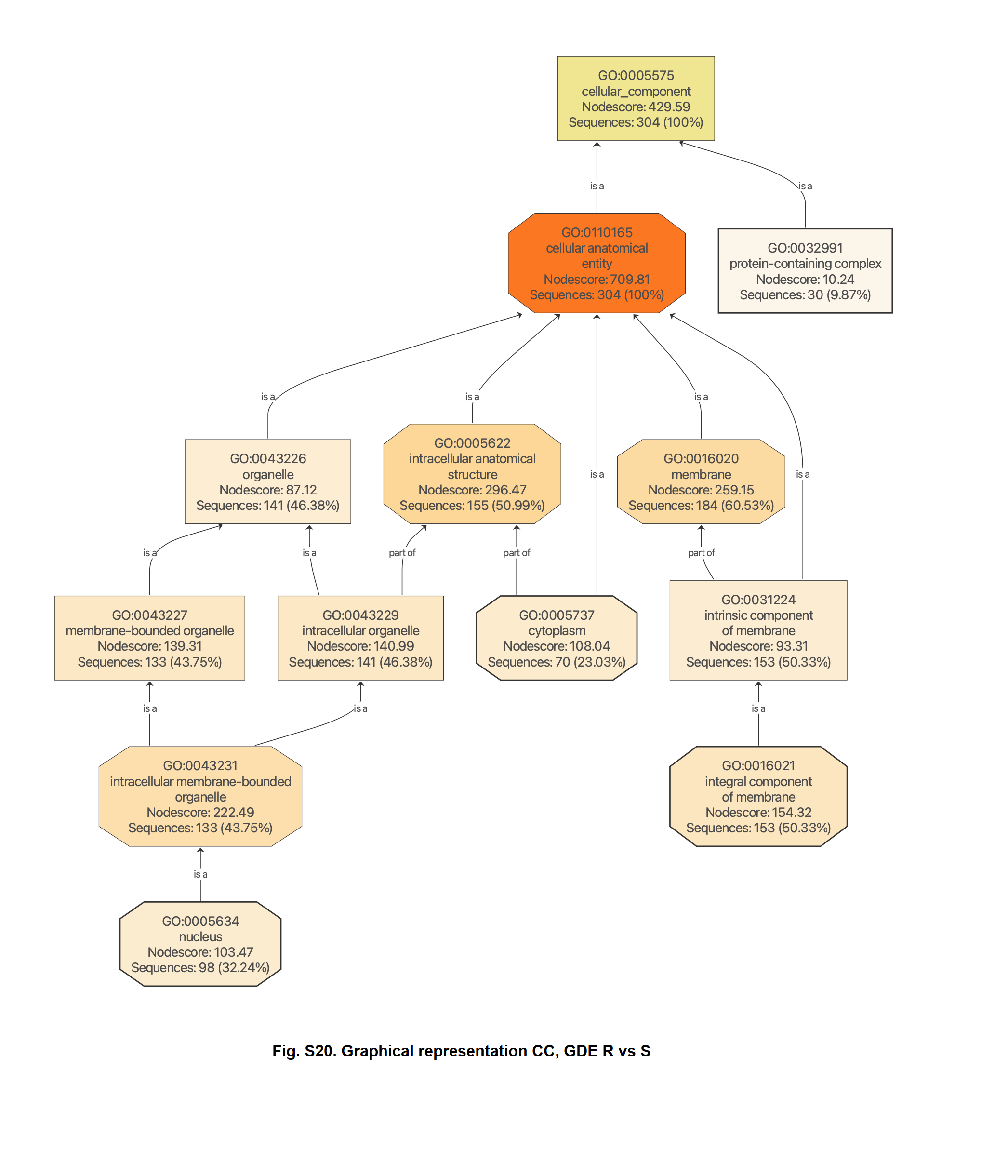

Supplement: Supplementary file 1 [file genes-13-00519-s001.zip › genes-1568159-supplementary/Supplementary Materials Final/Figure S20. Graphical representation CC, GDE R vs S.png]

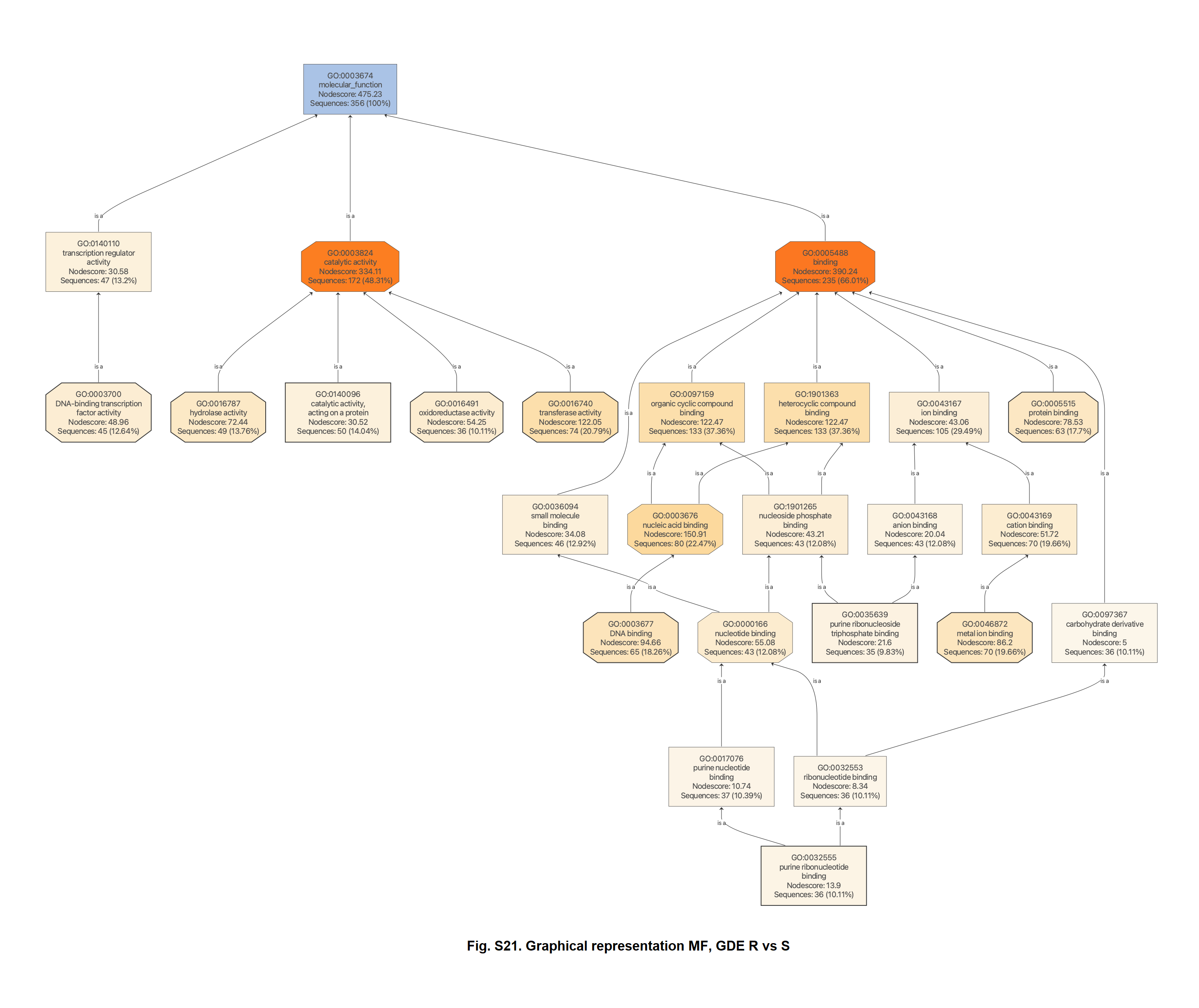

Supplement: Supplementary file 1 [file genes-13-00519-s001.zip › genes-1568159-supplementary/Supplementary Materials Final/Figure S21. Graphical representation MF, GDE R vs S.png]

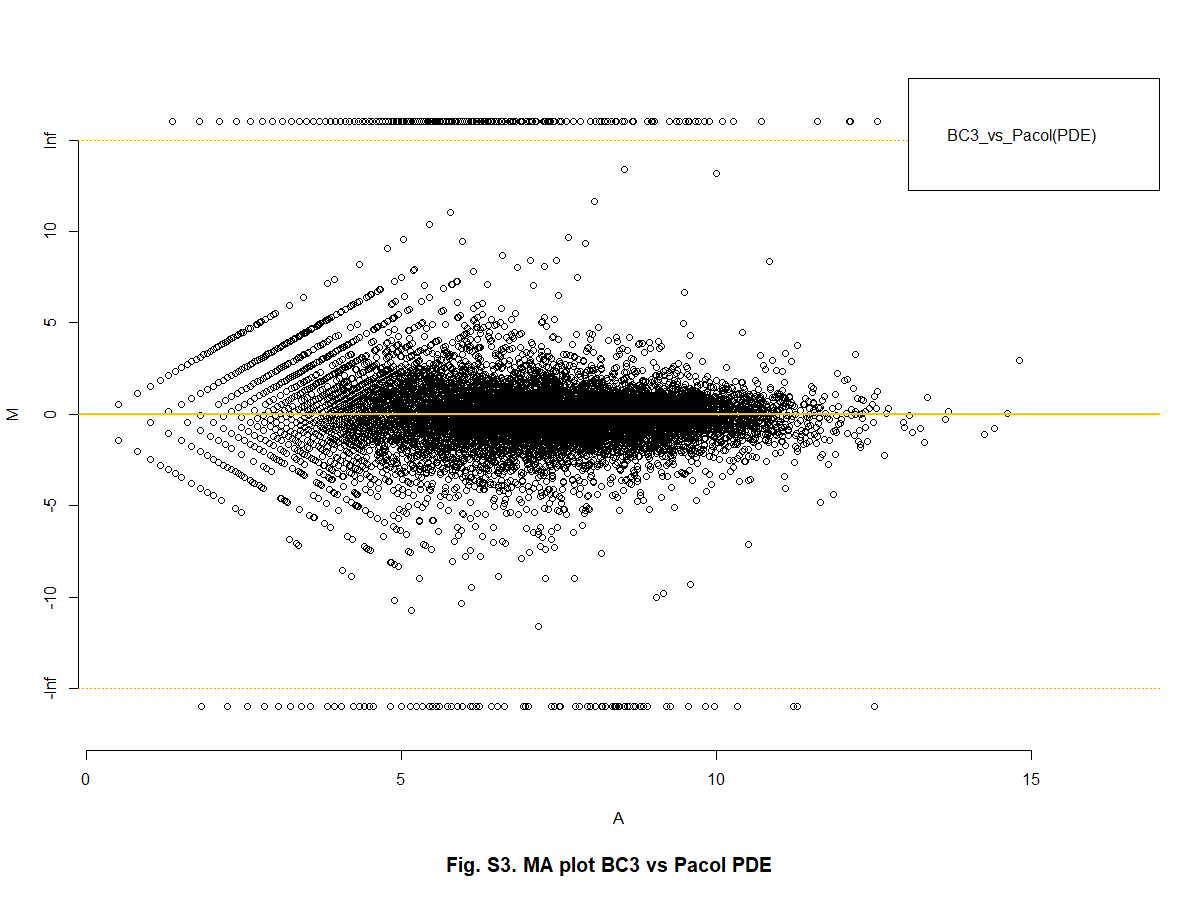

Supplement: Supplementary file 1 [file genes-13-00519-s001.zip › genes-1568159-supplementary/Supplementary Materials Final/Figure S3. MA plot BC3 vs Pacol PDE.png]

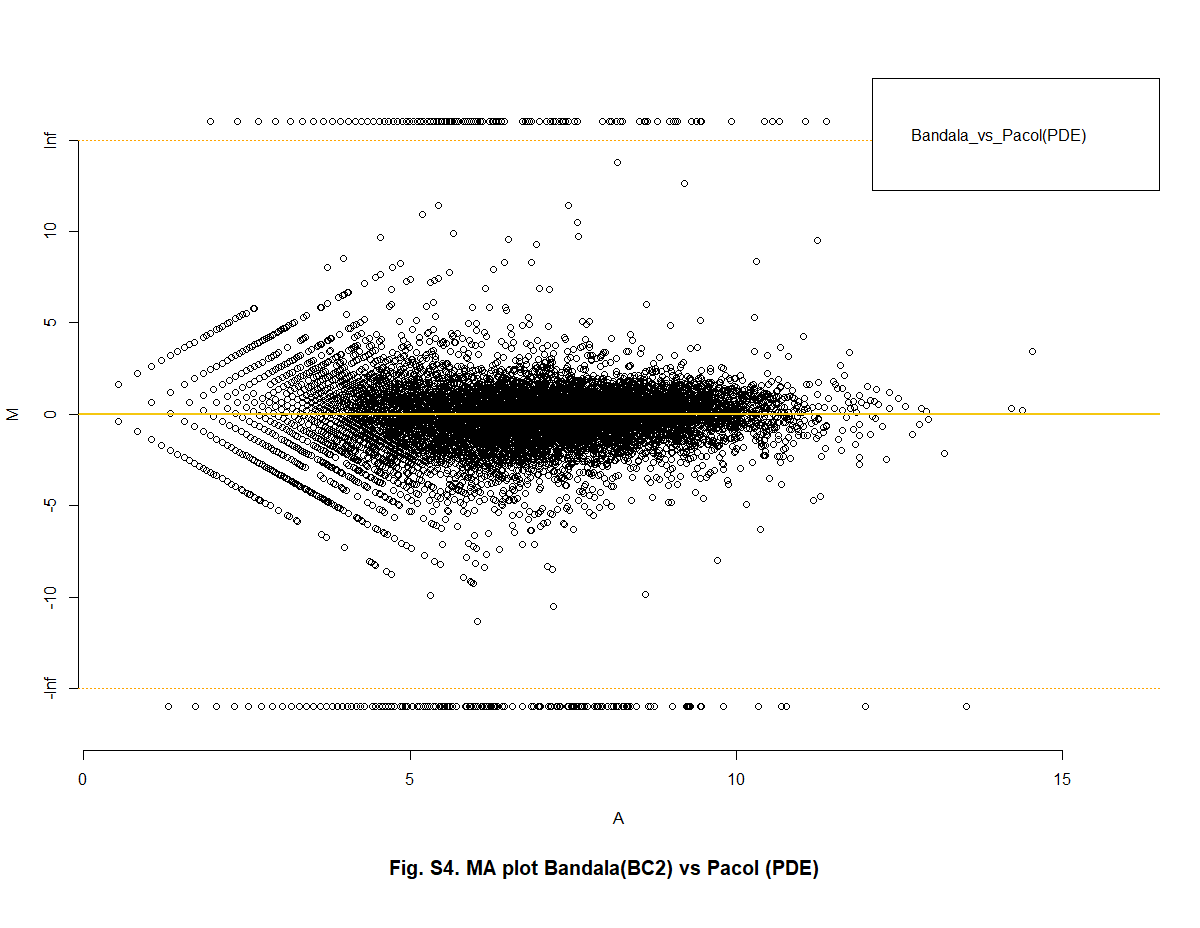

Supplement: Supplementary file 1 [file genes-13-00519-s001.zip › genes-1568159-supplementary/Supplementary Materials Final/Figure S4. MA plot Bandala(BC2) vs Pacol PDE.png]

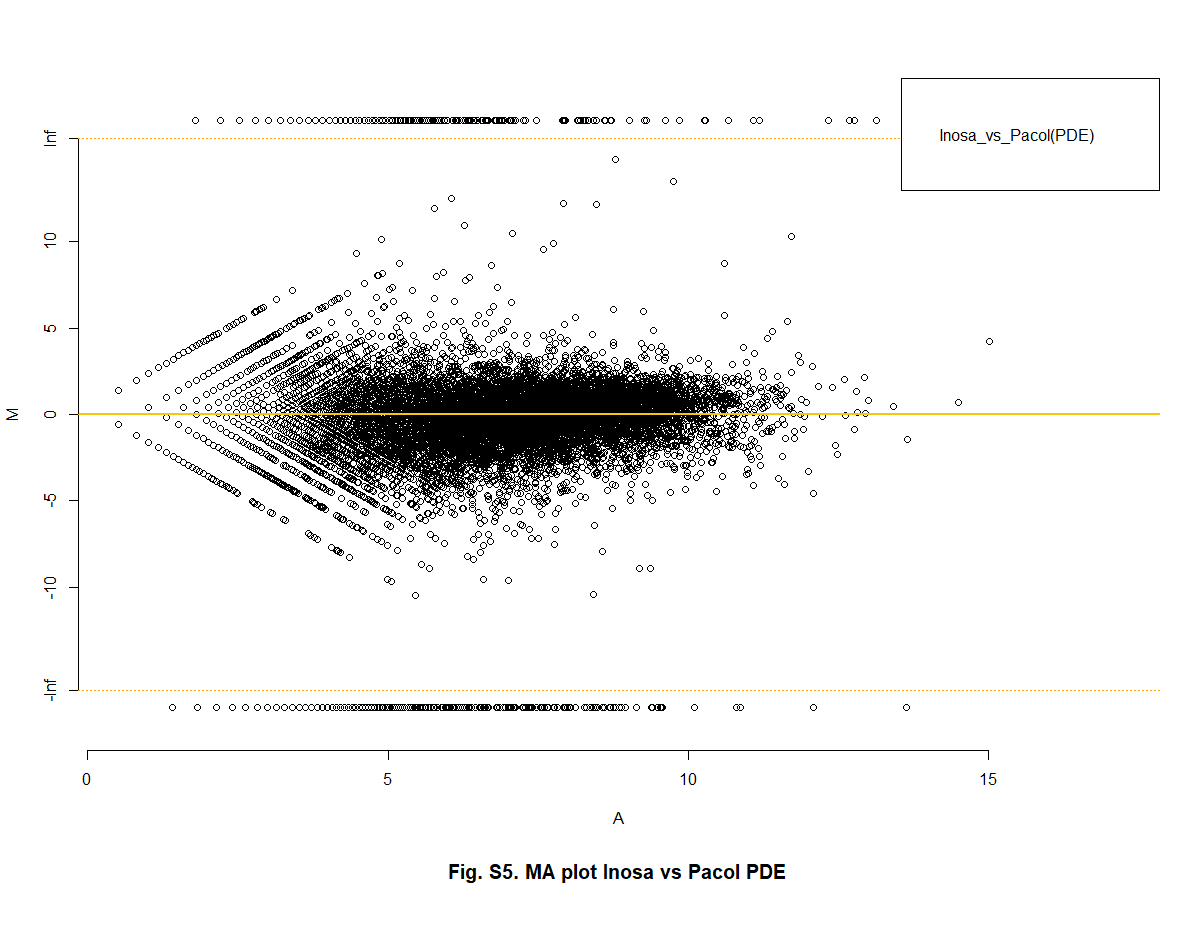

Supplement: Supplementary file 1 [file genes-13-00519-s001.zip › genes-1568159-supplementary/Supplementary Materials Final/Figure S5. MA plot Inosa vs Pacol PDE.png]

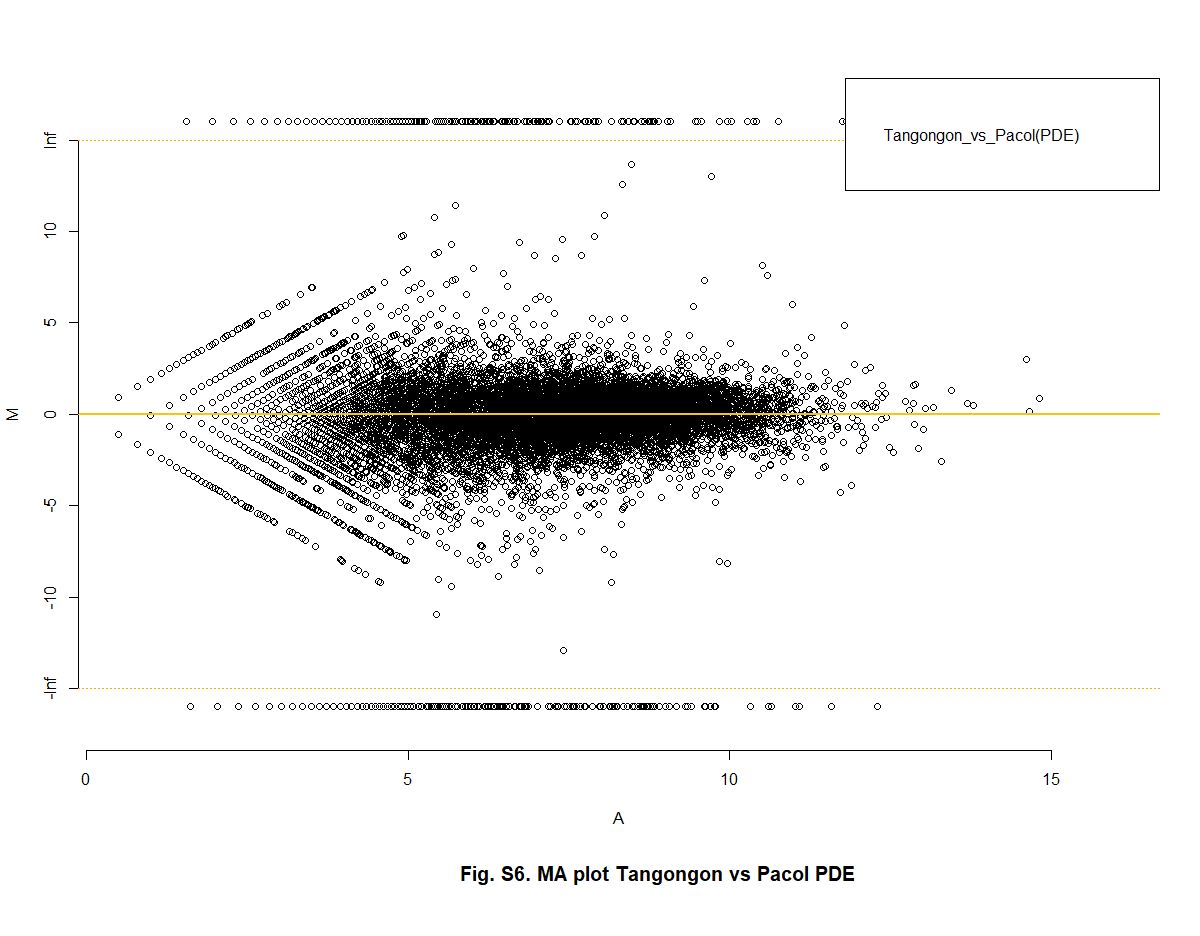

Supplement: Supplementary file 1 [file genes-13-00519-s001.zip › genes-1568159-supplementary/Supplementary Materials Final/Figure S6. MA plot Tangongon vs Pacol PDE.png]

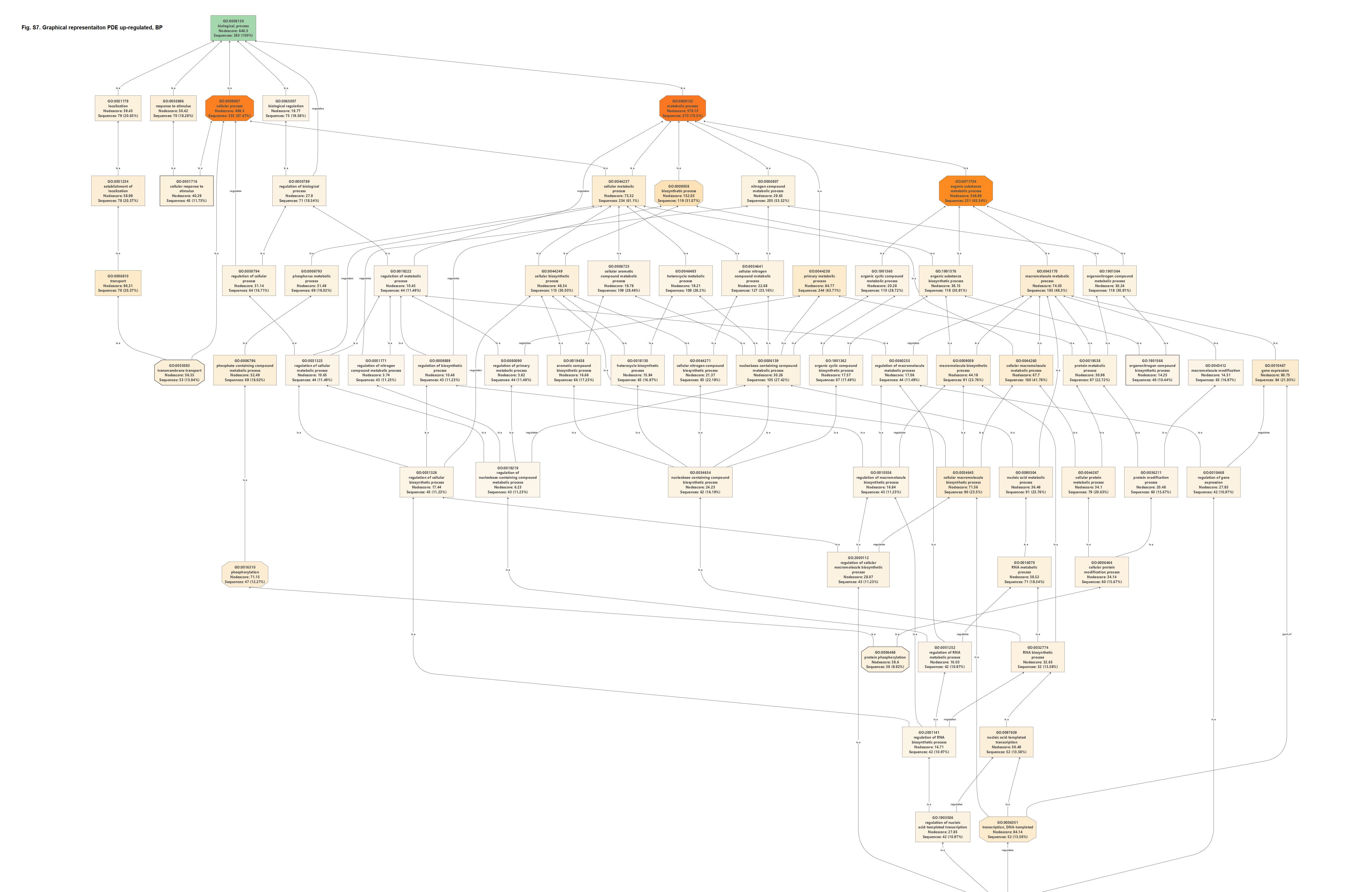

Supplement: Supplementary file 1 [file genes-13-00519-s001.zip › genes-1568159-supplementary/Supplementary Materials Final/Figure S7. Graphical representaiton PDE up-regulated, BP.png]

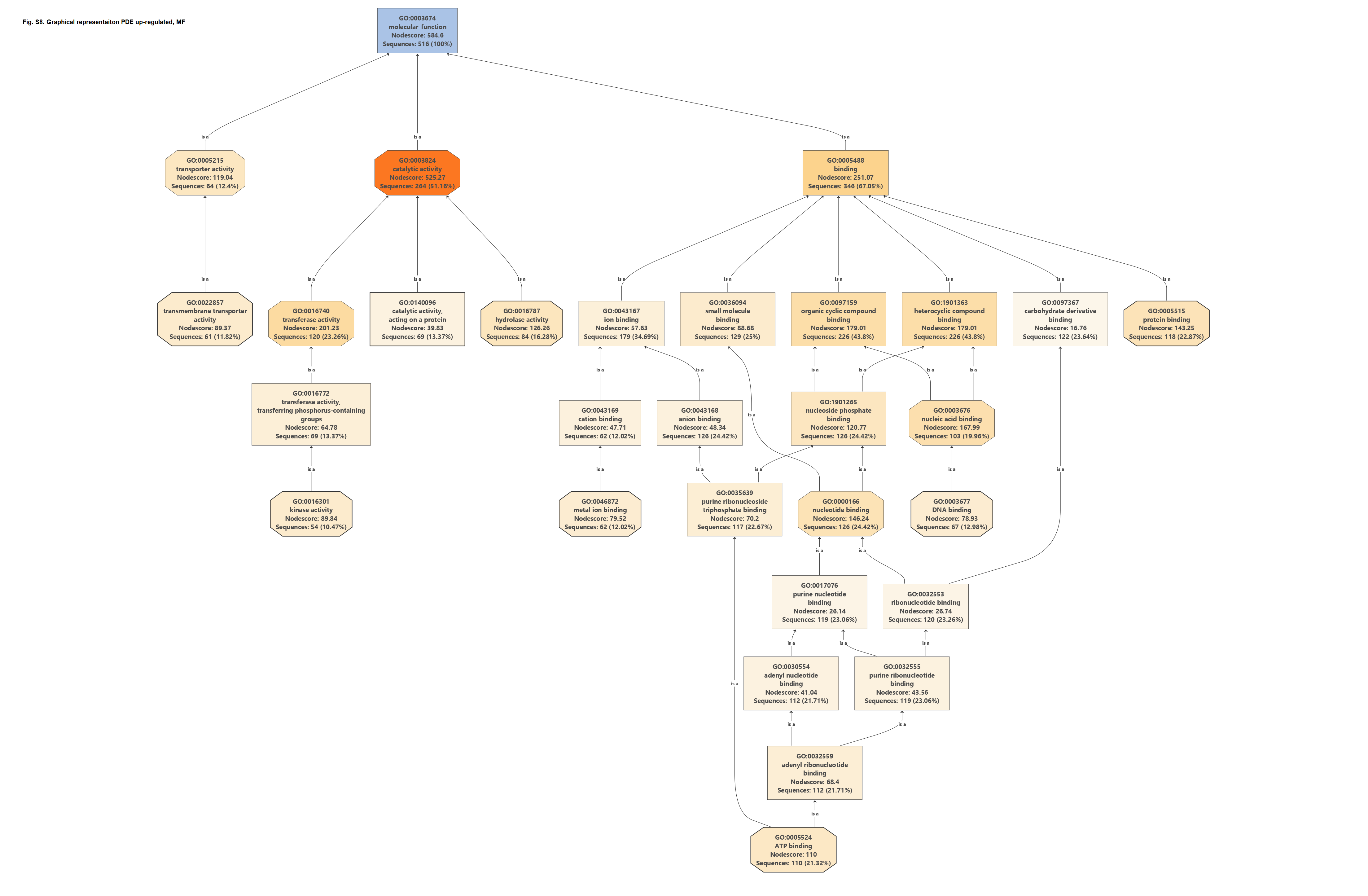

Supplement: Supplementary file 1 [file genes-13-00519-s001.zip › genes-1568159-supplementary/Supplementary Materials Final/Figure S8. Graphical representaiton PDE up-regulated, MF.png]

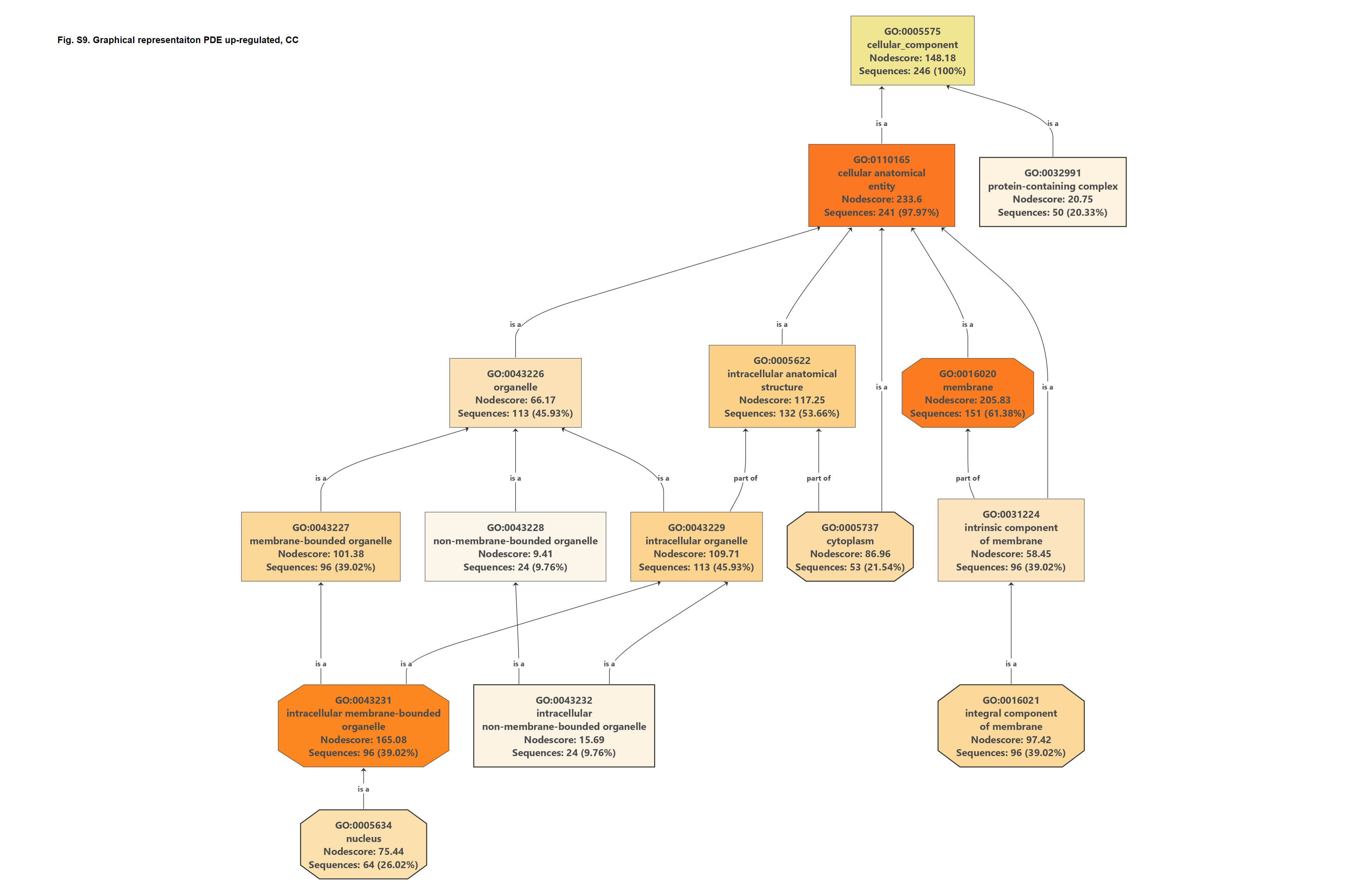

Supplement: Supplementary file 1 [file genes-13-00519-s001.zip › genes-1568159-supplementary/Supplementary Materials Final/Figure S9. Graphical representaiton PDE up-regulated, CC.png]
